# Exposure *in vivo* Induced Changes in Neural Circuitry for Pain-Related Fear: A Longitudinal fMRI Study in Chronic Low Back Pain

**DOI:** 10.3389/fnins.2019.00970

**Published:** 2019-09-17

**Authors:** Inge Timmers, Jeroen R. de Jong, Mariëlle Goossens, Jeanine A. Verbunt, Rob J. Smeets, Amanda L. Kaas

**Affiliations:** ^1^Department of Rehabilitation Medicine, Maastricht University, Maastricht, Netherlands; ^2^Department of Cognitive Neuroscience, Maastricht University, Maastricht, Netherlands; ^3^Department of Anesthesiology, Perioperative, and Pain Medicine, Stanford University, Palo Alto, CA, United States; ^4^Department of Rehabilitation Medicine, Maastricht University Medical Center, Maastricht, Netherlands; ^5^Adelante Centre of Expertise in Rehabilitation and Audiology, Hoensbroek, Netherlands; ^6^Department of Clinical Psychological Science, Maastricht University, Maastricht, Netherlands; ^7^CIR Revalidatie, Zwolle/Eindhoven, Netherlands

**Keywords:** chronic pain, exposure *in vivo*, neuroimaging, pain-related fear, rehabilitation, chronic low back pain

## Abstract

Exposure *in vivo* (EXP) is a cognitive-behavioral treatment aimed at reducing pain-related fear in chronic pain, and has proven successful in reducing pain-related disability in patients with chronic low back pain (cLBP). The current longitudinal study aimed to reveal the neural correlates of changes in pain-related fear as a result of EXP. Twenty-three patients with cLBP were included in this study. Patients with cLBP underwent MRI scanning pre-treatment (pre-EXP), post-treatment (post-EXP), and 6 months after end of treatment (FU-EXP). Pain-free controls were scanned at two time points. In the scanner, participants were presented with pictures involving back-related movements, evoking pain-related fear in patients. Pre-treatment, functional MRI revealed increased activation in right posterior insula and increased deactivation in medial prefrontal cortex (mPFC) in patients compared to controls. Post-treatment, patients reported reduced fear and pre-EXP group differences were no longer present. Contrasting pre- to post- and FU-EXP in patients revealed that stimulus-evoked neural responses changed in sensorimotor as well as cognitive/affective brain regions. Lastly, exploratory analyses revealed a tendency toward an association between changes in neural activation and changes in fear ratings, including the hippocampus and temporal lobe (pre- to post-EXP changes), and mPFC and posterior cingulate cortex (pre- to FU-EXP changes). Taken together, we show evidence that neural circuitry for pain-related fear is modulated by EXP, and that changes are associated with self-reported decreases in pain-related fear.

## Introduction

While most of us experience acute low back pain at some point in our lives, some will develop chronic low back pain (cLBP), with persistent pain lasting more than 6 months. An estimated one in five adults is currently in chronic pain, with cLBP being the most common (Breivik et al., 2006) and the world’s leading cause of disability (GBD 2015 Disease and Injury Incidence and Prevalence Collaborators, 2016; Hartvigsen et al., 2018). It is believed that maladaptive cognitions and emotional responses to pain are important factors for developing and maintaining chronic pain, as described by the fear avoidance model (Vlaeyen et al., 1995b, 2016). This model describes how, if immediate pain control is prioritized, pain-catastrophizing and pain-related fear may lead to pain-hypervigilance and avoidance behavior, and in turn increased functional disabilities. This then may amplify the pain experience and paradoxically increases pain-related fear, creating a vicious cycle. A subgroup of patients with cLBP indeed shows pain-related fears, including fear of movement and/or re-injury (Crombez et al., 1999; Vlaeyen and Crombez, 1999; Camacho-Soto et al., 2012; Thibodeau et al., 2013; Bunzli et al., 2015; Hartvigsen et al., 2018). In fact, pain-related fear is more closely linked to disability than pain intensity (Crombez et al., 1999; Zale et al., 2013).

To specifically target pain-related fears in clinical settings, Exposure *in vivo* (EXP) was developed. EXP is a cognitive-behavioral treatment based on experimental work showing that exposure to fearful activities and movements, rather than avoiding them, challenges catastrophic pain beliefs and can result in the extinction of fears and maladaptive responses (Vlaeyen et al., 1995a; Meulders and Vlaeyen, 2012). In EXP, movements and activities that are perceived as threatening and fearful are first identified using the pictorial tool The Photographic Series of Daily Activities (PHODA) (Leeuw et al., 2007). Then, the patient is repeatedly exposed to these feared movements and activities, while behavioral experiments are performed to challenge catastrophic expectations and interpretations regarding these movements, activities, and/or sensations. EXP has been applied as treatment for patients with chronic pain and elevated pain-related fear in a variety of settings and different pain conditions, including, but not limited to, non-specific cLBP. Ubiquitously, EXP has been successful in reducing pain-related fears and pain-related disability as compared to no treatment and at least as successful, if not more successful, in comparison to other treatments that are proven effective (Vlaeyen et al., 2001; Boersma et al., 2004; de Jong et al., 2005, 2008, 2012; Leeuw et al., 2008; Woods and Asmundson, 2008; den Hollander et al., 2016; Lalouni et al., 2016; Lopez-de-Uralde-Villanueva et al., 2016; Glombiewski et al., 2018).

It would be expected that EXP specifically impacts the neural circuitry involved in pain-related fear and fear extinction learning. Studies examining pain-related fear have identified altered neural responses in patients with cLBP to viewing and imagining activities/movements associated with pain (Taylor et al., 2015; Meier et al., 2016, 2017) – including increased recruitment of the insula, anterior cingulate cortex (ACC), amygdala, orbitofrontal cortex, striatum (i.e., regions involved in attentional/perceptual as well as affective/reappraisive aspects of pain), and altered crosstalk with the periaqueductal gray (PAG; involved in top-down pain modulation). For fear conditioning and extinction, experimental studies identified a core neural network, including the amygdala, insula, and ACC (Sehlmeyer et al., 2009; Fullana et al., 2016, 2018b). Only few imaging studies investigated fear learning and extinction in the context of pain (Kattoor et al., 2013; Labus et al., 2013; Icenhour et al., 2015), reporting altered neural responses in patients, including in the prefrontal cortex (PFC), ACC, insula, amygdala, hippocampus, PAG and thalamus. Further, results of clinical studies in chronic pain investigating treatment-induced functional brain changes show some overlap with neural changes related to pain-related fear and experimental fear extinction (e.g., implicating the amygdala, mPFC, and PAG) (Baliki et al., 2008; Becerra et al., 2014; Erpelding et al., 2014; Simons et al., 2014). The majority of treatment studies focused on intrinsic brain activity, i.e., in rest and without a specific task (Napadow et al., 2012; Harris et al., 2013; Bosma et al., 2018). The effects of EXP specifically have also only been investigated using resting-state fMRI (Zhu et al., 2018), showing that patients with post-traumatic stress disorder showed enhanced post-treatment resting-state functional connectivity between the amygdala, orbitofrontal cortex, hippocampus and the medial PFC. To date, there have been no studies investigating how (EXP) treatment modulates the circuitry underlying pain-related fear in chronic pain.

Therefore, the current longitudinal fMRI study tested the hypothesis that EXP acts upon the neural circuitry involved in pain-related fear, using a task designed to evoke pain-related fear. We compared patients with cLBP with pain-free volunteers pre- and post-EXP treatment; the cLBP group was also examined 6 months after end of treatment. We evaluated group differences and treatment effects in evoked brain activation. Also, more exploratively, we used (changes in) fear ratings to identify neural correlates specific to (reductions in) pain-related fear. A whole-brain approach was adopted in combination with analyses in *a priori* defined regions of interest (ROIs) that were considered to be of particular interest due to their involvement in pain-related fear and experimental extinction learning (i.e., amygdala, hippocampus, mPFC, PAG) and/or pain chronification (i.e., mPFC, NAc). We expected (I) pre-treatment group differences in neural circuitry recruited by stimuli evoking pain-related fear, correlated to fear ratings as well as pain-related outcomes in patients; (II) patient-specific pre- to post-treatment changes in regions showing pre-treatment group differences, as well as in other brain regions associated with chronic pain and with extinction (i.e., amygdala, hippocampus, mPFC, NAc, PAG); (III) pre- to post-treatment changes associated with changes in fear and persisting at 6 months follow-up.

## Materials and Methods

### Overall Study Procedure

This study presents data of a larger study investigating effects of EXP on chronic pain, “BrainEXPain”. BrainEXPain was approved by the Medical Ethical Committee of Maastricht University Hospital/Maastricht University (MUMC+/UM), and the protocol is registered at ClinicalTrials.gov [NCT02347579]. Patient recruitment was done via the department of Rehabilitation Medicine at MUMC+/Adelante rehabilitation center where patients were seen for consultation. If patients were found motivated for rehabilitation treatment and eligible for the multi-disciplinary pain screening program, they were invited by the physiatrist for the study. Recruitment was open between January 2015 and August 2017.

Participants were then contacted by the research team and were screened for in- and exclusion criteria. Informed consent was obtained at study enrollment. Prior to scanning, all participants filled in questionnaires online (Qualtrics, Provo, United States^1^). The first study visit was scheduled prior to any (information on) treatment (i.e., baseline or pre-EXP). Afterward, patients underwent a multi-disciplinary pain screening and pain education, and started the exposure sessions (if eligible for treatment) – which were all part of standard care. At the end of treatment, patients underwent a post-EXP and a follow-up study visit (6 months after end of treatment; FU-EXP). Healthy controls participated in two study visits, with the time in between these visits matching the patients’ pre- to post-EXP. Participants received €15 per study visit and travel reimbursement for their participation.

### Participants

Inclusion criteria for patients were age between 18 and 65 years, stable medication,^2^ experience of non-specific LBP > 6 months, and no other diagnosis explaining the symptoms. Exclusion criteria were claustrophobia, MRI incompatibility (e.g., pacemaker, pregnancy), and severe psychopathology (Symptom Check List-90). Of the 35 patients with cLBP invited by the physiatrist over the 2.5 years inclusion period, 23 patients with cLBP were included in BrainEXPain (8 patients were not interested in participating, 4 patients were MRI incompatible). Of these, three patients dropped out prior to or during the measurement (due to claustrophobia); of two patients the data analyzed here was not acquired due to technical error; three patients were excluded due to extensive motion (see Data Analysis); and one patient was excluded due to lack of any vision-related (occipital) activity (see Data Analysis). The final sample for this study therefore consisted of 14 patients (Table 1). Post-EXP data is available for 10 patients (three did not start EXP, one became MRI incompatible), and FU-EXP data is available for 9 patients (1 was lost to follow-up due to unrelated medical issues).

**TABLE 1:** Demographics of the final sample.

	**Patients with cLBP**	**Pain-free volunteers**	**Statistics for group**
	**Mean (SD)**	**Mean (SD)**	**comparison**
Sample size	*n* = 14	*n* = 14	n.a.
Age (years)	42.4 (11.6)	41.7 (12.5)	*F*_(1,26)_ = 0.02, *p* = 0.89
Sex	11 males 3 females	10 males 4 females	*X*^2^_(1,*n* = 28)_ = 0.19, *p* = 0.66
Handedness	13 right-handed 0 left-handed 1 ambidextrous	14 right-handed 0 left-handed 0 ambidextrous	*X*^2^_(1,*n* = 28)_ = 1.04, *p* = 0.31
Pain duration	6–12 months: *n* = 1 1–2 years: *n* = 3 2–5 years: *n* = 8 >5 years: *n* = 2	n.a.	n.a.

**n.a., not applicable.**

The patient group was compared to a sample of 14 pain-free healthy volunteers, matched for age, sex and handedness on cohort-level. To match the patient group, 10 controls underwent a second study visit. Controls were recruited through local advertisements. Additional exclusion criteria were: history of a chronic pain syndrome, and seeking treatment for a pain condition in the last 6 months.

### Exposure *in vivo* Treatment

Within MUMC+/Adelante, EXP is standard care for patients with cLBP presenting with elevated pain-related fear. No additional restrictions or requirements for EXP were set by BrainEXPain. EXP specifically aims to reduce disability by challenging erroneous interpretations and expectancies about pain (e.g., that pain always indicates harm or that activities cause harm). A detailed description of the exposure-protocol for pain-related fear can be found in Vlaeyen et al. (2012). In brief, EXP always started with identifying movements/activities that are perceived as threatening and fearful, education about treatment rationale and that harm or pain does not mean additional injury (i.e., by discussing MR images of the spine by the treating physiatrist). EXP then continued with repeated exposure to feared movements, activities and/or sensations combined with behavioral experiments to challenge catastrophic interpretations by creating violations of expectancies. Patients were furthermore instructed to keep performing the movements and/or activities they performed during their sessions. EXP typically consists of 16 sessions (although it could be shortened to 8 or extended to 20, per clinicians’ decision), which are guided by a psychologist and either a physical or an occupational therapist. To identify movements and activities that are perceived as threatening and fearful, EXP utilizes The Photographic Series of Daily Activities (PHODA) for the low back (Leeuw et al., 2007). The PHODA consists of photographs depicting back-related movements and activities that are rated based on their perceived harmfulness. See Table 2 for more participant and EXP-related characteristics.

**TABLE 2:** Information about EXP and the repeated measures.

	**Patients with cLBP**	**Pain-free volunteers**	**Statistics for group**
	**Mean (SD)**	**Mean (SD)**	**comparison**
Sample size	*n* = 10 (*n* = 9 for FU-EXP)	n = 10	n.a.
Age (years)	40.2 (11.3)	39.6 (12.2)	*F*_(1,18)_ = 0.01, *p* = 0.91
Sex	9 males 1 female	8 males 2 females	*X*^2^_(1, *n* = 20)_ = 0.39, *p* = 0.53
Pain duration	6–12 months: *n* = 1 1–2 years: *n* = 2 2–5 years: *n* = 5 >5 years: *n* = 2	n.a.	n.a.
EXP treatment duration (days)	45.0 (15.9)	n.a.	n.a.
Time between pre-EXP session and start EXP treatment (days)	29.3 (12.2)	n.a.	n.a.
Time between pre-EXP and post-EXP session (days)	96.1 (42.1)	92.3 (33.5)	*F*_(1,18)_ = 0.05, *p* = 0.83
Time between post-EXP and FU-EXP session (days)	186.4 (9.6)	n.a.	n.a.

**n.a., not applicable.**

### Assessment of Pain-Related Aspects and Performance Levels

At all time-points we assessed: pain intensity using a 0–10 visual analog scale anchored with “no pain at all” and “worst pain imaginable”; pain-related fear using the PHODA short electronic version for low back (Leeuw et al., 2007), and Tampa Scale for Kinesiophobia (TSK; Kori, 1990; Vlaeyen et al., 1995a), Pain Catastrophizing Scale (PCS; Sullivan et al., 1995; Crombez et al., 1999), Pain Disability Index (PDI; Tait et al., 1987; Soer et al., 2013), Physical Activity Rating Scale combined with the Perceived Activity Decline (PARS/PAD; Vercoulen et al., 1997; Verbunt, 2008) questionnaire. Only assessed at baseline as trait measures were: Fear of Pain Questionnaire (PFQ; McNeil and Rainwater, 1998; van Wijk and Hoogstraten, 2006) and State Trait Anxiety Inventory (STAI-Y2; van der Ploeg et al., 1980; Spielberger et al., 1983). In addition, all participants underwent performance testing during all study visits to assess functioning. In the *2 min walking test*, participants walked for 2 min on a standardized track and the covered distance was measured in meters. During *staircase walking*, participants walked a complete staircase (up and down), after which the average time per step was calculated.

### Picture Imagination Task

In the scanner, the participants were presented with visual stimuli, associated with one of three categories: rest (derived from a web-search – REST), movements and activities perceived as fearful for patients specifically (derived from the extended version of the PHODA, not used in pain assessment and/or treatment – MOVEMENT), or pictures implying bodily damage that may be perceived as fearful in general (derived from IAPS (Lang et al., 1997) and a web-search – MEDICAL). Backgrounds were removed to make the physical properties as similar as possible.

**FIGURE 1:**
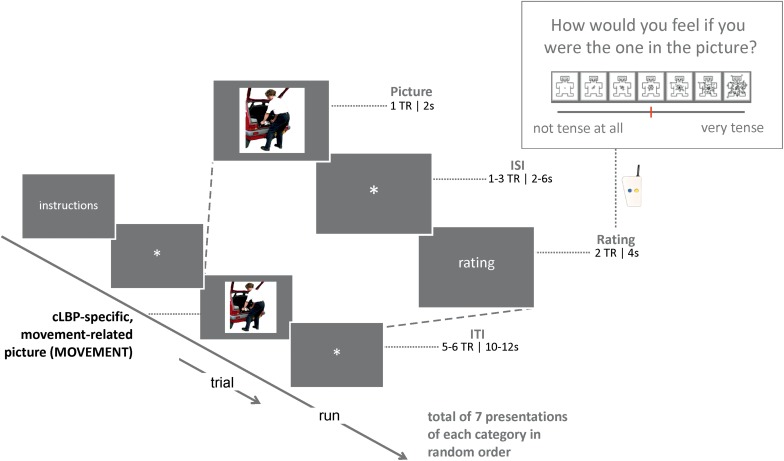
Schematic overview of the design of the picture imagination task, showing the elements plus corresponding timing and a zoom into one example trial; TR, repetition time; ISI, inter-stimulus time; ITI, inter-trial time.

Participants were instructed to carefully look at the pictures and imagine that they were the person in the picture (carrying out the movement or activity, if applicable). After a short delay (see Figure 1 for details), participants were asked to rate how they would feel if they were the person on the picture (indirect assessment of fear). Ratings were done by pressing a button that moved a cursor on a horizontal line presented on the screen (later converted to 0–10 scores). In total, there were 21 trials (7 of each category). Stimuli were presented using Presentation Software (Neurobehavioral Systems Inc.), and were synchronized with MR data acquisition. The total task had a duration of approximately 8 min. The picture imagination task was always performed second, after a resting-state run. The total duration of the scan was approximately 75–90 min (data from other runs will be described elsewhere).

### MRI Acquisition

MRI data were collected using a 3 Tesla whole body MRI scanner (Philips Gyroscan Achieva TX) using a 32-channel head coil, at the department of Radiology at MUMC+.

For the functional images, a T2^∗^-weighted standard echo-planar imaging (EPI) sequence was used to acquire 40 axial slices (3 mm isotropic) covering the entire cortical volume, using the following parameters: repetition time (TR) = 2000 ms, echo time (TE) = 25 ms, flip angle = 75°, matrix size = 120 × 240, SENSE factor = 2. In total, 225 functional volumes were collected, of which the first two volumes were dummy volumes that were discarded from subsequent analysis to avoid T1 saturation effects.

T1-weighted anatomical images were acquired using a 3D turbo field echo (TFE) sequence with the following parameters: 170 slices, 1 mm isotropic, TR = 8.1 ms, TE = 3.7 ms, flip angle = 8°, matrix size = 240 × 240.

### Data Analysis

#### Assessment of Pain-Related Outcomes

Questionnaire and performance test data were analyzed using SPSS (version 24). A general linear model (GLM) with Group (patients, controls) as between-subjects (BS) factor was used to examine group differences pre-EXP, as well as post-EXP. In addition, a repeated measures (rmGLM) with Time [pre-EXP, post-EXP, (FU-EXP)] as a within-subjects (WS) factor was used to investigate changes over time.

#### Behavioral Data: Picture Imagination Task

Group comparisons in in-scanner fear ratings, focusing on MOVEMENT pictures, were evaluated using a rmGLM with Group (patients, controls) as BS factor and Picture Number (7 different Pictures per Category) as WS factor. In addition, the WS factor Time [pre-EXP, post-EXP, (FU-EXP)] was added in a separate analysis.

#### MRI Data: Pre-processing

MRI data analysis was performed using BrainVoyager 3.6 (Brain Innovation, Maastricht, the Netherlands). Pre-processing of the functional data included slice scan time correction, 3D head motion correction, linear trend removal, high-pass filtering (5 cycles per run; corresponding to 0.1 Hz), and spatial smoothing [4 mm using a full-width at half-maximum Gaussian kernel (FWHM)]. Data was then co-registered to the corresponding anatomical image, and normalized to MNI space. The three pictures categories (REST, MOVEMENT, and MEDICAL) plus the delay prior to the rating (i.e., in total 4–8 s) were used as predictors, convolved with the hemodynamic response function (HRF). Additional information on denoising procedures can be found in Supplementary Information.

#### MRI Data Analysis: Masking

Whole-brain analyses were run within a mask that excluded the white matter and cerebral spinal fluid, based on the Harvard-Oxford atlases (probability threshold 0.25) (Frazier et al., 2005; Desikan et al., 2006; Makris et al., 2006; Goldstein et al., 2007). To specifically test our hypotheses in brain regions that play important roles in chronic pain and/or fear extinction, additional analyses were run within predefined region-of -interest (ROI) masks. ROIs were defined in bilateral medial frontal cortex (mPFC), bilateral amygdala, bilateral nucleus accumbens (NAc), bilateral hippocampus based on the Harvard-Oxford subcortical atlas (probability threshold 0.25). A ROI corresponding to bilateral PAG was defined by dilating spheres around coordinates from Linnman et al. (2012) [*x* = 1, *y* = –29, *z* = –10 (volume = 1612 mm^3^, diameter ∼14.5 mm)]. In these ROI masks, FDR correction [*q*(FDR) < 0.05] and minimum cluster size of 4 voxels (108 mm^3^) was used for statistical thresholding.

#### MRI Data: Group Differences and Treatment Effects

To compare blood-oxygen-level dependent (BOLD) responses across Groups and Times, a univariate random-effects (RFX) analysis with separate subject predictors was run at the first level, after which this data was fed into a second-level RFX analysis where group maps could be estimated and contrasted. FDR correction [*q*(FDR) < 0.05] was used for map creation. In the whole-brain analysis, an initial threshold of *p* < 0.001 was used for contrasts across Groups and Times, after which cluster-size thresholding was performed using MonteCarlo simulations (*n* = 1000) to correct maps at the level of alpha 0.05. The main contrast of interest was MOVEMENT vs. baseline, plus effects of Group and Time herein, as this condition was designed to elicit pain-related fear specifically in the patient group.

#### MRI Data: Correlations With (Changes in) Pain-Related Outcome Measures and Changes in Fear

Two types of correlation analyses were performed. From regions in which significant Group and Time differences were observed, betas were extracted in order to perform correlation analyses with measures of pain-related outcomes. An additional, explorative, analysis for the patients was to examine correlations between changes in fear ratings and changes in neural activation patterns at a whole-brain level. For this, we used the percentage of change in fear ratings for MOVEMENT pictures (at post- and FU-EXP compared to pre-EXP), and took a less conservative initial cluster-defining threshold of *p* < 0.005 for the cluster-size thresholding.

## Results

### Pre-treatment (Pre-EXP) Data

#### Patients Show High Levels of Fear, Pain, and Disability Pre-EXP

Pre-treatment, patients reported significantly higher levels of pain, pain-related fear, catastrophizing and disability compared to controls (Table 3). Groups furthermore differed in trait anxiety, but not in trait fear of pain. Also, patients reported significantly lower levels of physical activity and higher levels of perceived activity decline compared to controls. Lab-assessed performance tests confirmed this: patients covered significantly less distance within 2 min walking, and needed more time to walk stairs, compared to controls.

**TABLE 3:** Self-reported measures and performance tasks at baseline (pre-EXP).

	**Patients with cLBP Mean (SD)**	**Pain-free volunteers Mean (SD)**	**Statistics for group comparison**
**Self-reported measures**			
Pain intensity (VAS) (range 0–10)	5.5 (2.4)	0.2 (0.5)	*F*_(1,26)_ = 64.39, *p* < 0.001^∗∗^, η*_*p*_*^2^ = 0.71, 95% CI = 3.9, 6.7
Pain-related fear (PHODA) (range 0–100, cutoff score 38)	55.0 (23.9)	2.3 (3.5)	*F*_(1,26)_ = 66.84, *p* < 0.001^∗∗^, η*_*p*_*^2^ = 0.72, 95% CI = 39.5, 66.0
Fear of movement (TSK) (range 17–68)	40.9 (9.0)	27.4 (4.8)	*F*_(1,26)_ = 26.33, *p* < 0.001^∗∗^, η*_*p*_*^2^ = 0.48, 95% CI = 7.8, 19.0
Pain catastrophizing (PCS) (range 0–52, cutoff score 21)	24.2 (14.2)	3.6 (3.7)	*F*_(1,26)_ = 27.71, *p* < 0.001^∗∗^, η*_*p*_*^2^ = 0.52, 95% CI = 12.5, 28.6
Pain disability (PDI) (range 0–70)	39.6 (15.9)	1.9 (5.1)	*F*_(1,26)_ = 67.04, *p* < 0.001^∗∗^, η*_*p*_*^2^ = 0.73, 95% CI = 28.3, 47.3
Perceived activity decline (PAD) (range 0–20)	12.1 (7.4)	0.1 (0.5)	*F*_(1,26)_ = 36.53, *p* < 0.001^∗∗^, η*_*p*_*^2^ = 0.58, 95% CI = 8.0, 16.1
Physical activity (PARS) (range 0–100)	35.6 (6.7)	45.4 (4.6)	*F*_(1,26)_ = 19.51, *p* < 0.001^∗∗^, η*_*p*_*^2^ = 0.43, 95% CI = –13.8, –5.0
Trait anxiety (STAI-Y2) (range 20–80)	42.6 (10.5)	31.4 (5.3)	*F*_(1,26)_ = 21.02, *p* < 0.001^∗∗^, η*_*p*_*^2^ = 0.46, 95% CI = 8.2, 21.5
Trait fear of pain (FPQ) (range 0–150)	47.6 (10.4)	55.8 (15.2)	*F*_(1,26)_ = 2.71, *p* = 0.11, η*_*p*_*^2^ = 0.10, 95% CI = –18.5, 2.1
**Performance tasks**			
Two-min walking test (distance in meters)	148.1 (48.4)	236.9 (28.3)	*F*_(1,25)_ = 34.53, *p* < 0.001^∗∗^, η*_*p*_*^2^ = 0.58, 95% CI = –119.9, –57.7
Stair case walking (average time per step in seconds)	1.52 (0.13)	0.62 (0.04)	*F*_(1__,__25__)_ = 19.32, *p* < 0.001^∗∗^, η*_*p*_*^2^ = 0.45, 95% CI = 0.48, 1.33

**^∗∗^Survives Bonferroni correction for multiple comparison (alpha = 0.05/11 = 0.0045); size; 95% CI = 95% confidence interval of the difference (patients – controls).**

#### Patients Report More Fear for MOVEMENT Pictures Pre-EXP

**FIGURE 2:**
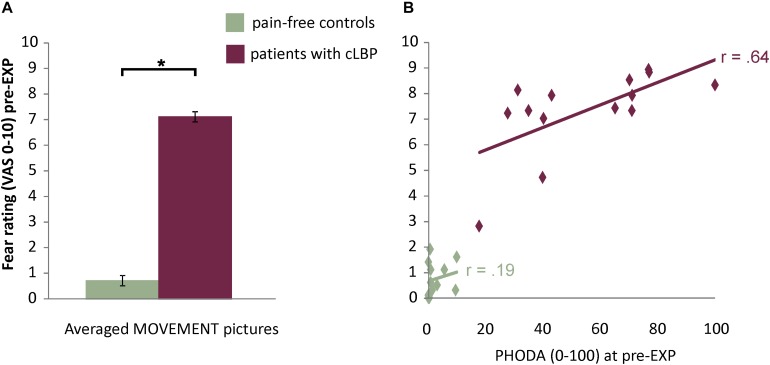
**(A)** Fear ratings for the MOVEMENT pictures. Presented are means and standard errors for each group. Horizontal lines and asterisks indicate significant effects (^∗^*p* < 0.05). **(B)** Correlation between fear ratings for MOVEMENT pictures and pain-related fear as assessed using the PHODA.

The in-scanner fear ratings for MOVEMENT pictures showed a significant Group effect [*F*_(1,26)_ = 188.15, *p* < 0.001, η*_*p*_*^2^ = 0.88, 95% CI = 5.6, 7.5], where patients reported higher fear levels compared to controls (Figure 2 and Supplementary Figure S1 for fear ratings for all Picture Categories). Also, for patients, fear ratings were significantly and strongly correlated with pain-related fear as assessed using the PHODA (*r* = 0.64, *p* = 0.01) (Figure 2).

#### Patients Show Increased BOLD Activation to MOVEMENT Pictures Pre-EXP

**FIGURE 3:**
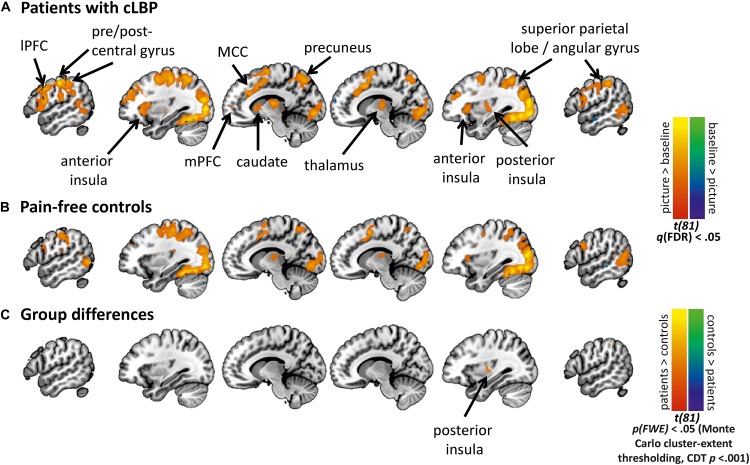
Activation maps for the MOVEMENT Picture Category at pre-EXP, per group. Statistical maps are presented showing the neural activation of the MOVEMENT category relative to baseline for **(A)** Patients with cLBP, and **(B)** Pain-free volunteers, **(C)** Group differences in MOVEMENT condition. Cluster-level correction using *p* < 0.001 as initial threshold. CDT, cluster-defining threshold. lPFC, lateral prefrontal cortex; mPFC, medial prefrontal cortex; MCC, mid-cingulate cortex.

Figure 3 shows activation maps for the MOVEMENT pictures, per Group (see Supplementary Figure S2 for activation maps of all Picture Categories). Overall, the MOVEMENT pictures elicited activation in a similar network in patients and controls.

**FIGURE 4:**
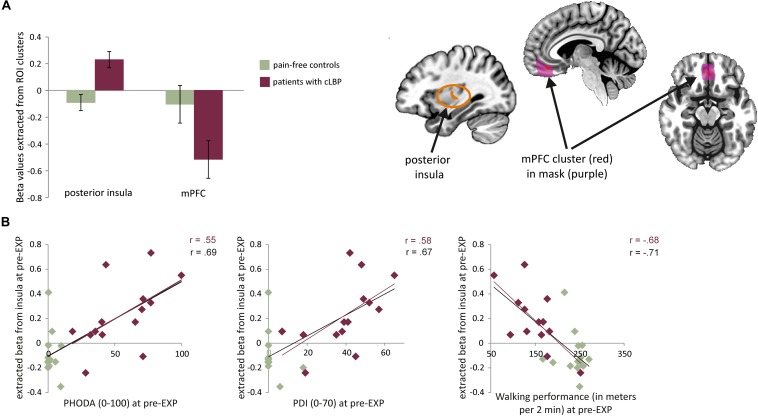
**(A)** Left: average beta values and standard errors for the MOVEMENT vs. baseline contrast for each group at pre-EXP, extracted from the two areas showing group differences. Right: Depiction of the location of the identified clusters. **(B)** Correlations between the posterior insula activation (beta value) and pain-related variables (self-reported and performance tasks). Note that the trendlines and magnitude of the correlations are shown for both the whole group (black) as well as the patient group only (red). mPFC, medial prefrontal cortex; PDI, pain disability index.

The whole-brain analysis showed a significant group difference in the right posterior insula (MNI *x* = 33, *y* = −10, *z* = 10, *k* cluster size = 206 mm^3^), with patients showed increased BOLD activation compared to controls (Figures 3, 4A). The masked analyses in the pre-defined ROIs additionally showed a difference in mPFC (MNI *x* = 0, *y* = 41, *z* = −11, *k* = 4 mm^3^), with patients showing increased BOLD deactivation compared to controls (Figure 4A, Supplementary Figure S2, and Supplementary Tables S1, S2).

#### Patients’ Neural Activation to MOVEMENT Pictures Shows Specific Correlation to Pain-Related Outcomes

Correlation analyses were performed using betas extracted from the right posterior insula and mPFC (i.e., averaged across all voxels in the cluster). When investigating the entire sample, both the activation in the posterior insula and mPFC was correlated to pain intensity and pain-related fear. Activation in the posterior insula was furthermore correlated to pain catastrophizing, pain disability, and both performance tests (Table 4). When zooming into the patient group, activation during MOVEMENT pictures in the posterior insula was positively correlated with pain-related fear, pain disability and both performance tasks while activation in mPFC did not correlate with any of the variables (Table 4). For the posterior insula, correlations reflected that increased neural activation was related to increased levels of fear, disability and worse performance (Figure 4B). For the mPFC, the correlations were negative and reflected that decreased neural activation was related to increased levels of fear.

**TABLE 4:** Correlations at pre-EXP between pain-related variables and activation in the regions displaying a group difference.

	**Right posterior insula**		**mPFC**	
	**Whole group**	**Patients only**	**Whole group**	**Patients only**
Pain-related fear (PHODA)	0.69^∗∗^	0.55^∗^	–0.43^∗^	–0.23
Fear of movement (TSK)	0.53^∗∗^	0.40	–0.39^∗^	–0.14
Pain catastrophizing (PCS)	0.51^∗∗^	0.27	–0.23	–0.03
Pain disability (PDI)	0.67^∗∗^	0.58^∗^	–0.38	–0.13
Pain intensity (VAS)	0.56^∗∗^	0.23	–0.48^∗^	–0.36
Walking performance	–0.71^∗∗^	–0.68^∗^	0.31	0.25
Staircase walking performance	0.73^∗∗^	0.71^∗^	–0.32	–0.20
Trait fear of pain (FPQ)	–0.33	0.14	0.33	0.39
Trait anxiety (STAI-Y2)	0.48^∗^	0.36	–0.34	–0.14

**^∗∗^p < 0.005 (Bonforroni correction for multiple comparison = 0.05/9 = 0.0055), ^∗^p < 0.05, whole group = patients and controls together n = 28, patients only n = 14.**

### Effects of Exposure *in vivo* Treatment

#### Patients Show Improvements in Fear and Functioning After EXP Treatment

##### Pre- to post- to FU-EXP changes in patients

Patients showed main effects of Time for pain-related fear, pain-related disability, perceived activity decline, and the performance tests (Table 5). There were no main effects for pain intensity, pain catastrophizing and self-reported physical activity, although these measures generally showed a decrease, and showed clinically relevant reductions (defined as reduction of 30% or more compared to baseline) in 60, 60, and 40% of patients in these domains, respectively, from pre- to post-EXP (Table 5).

**TABLE 5:** EXP-induced changes in self-reported measures and performance tasks in the patient group.

	**Pre- to post-EXP change mean (SE)**	**Pre- to FU-EXP change mean (SE)**	**Stats main effect Session**	***Post hoc* comparisons**	**> 30% reduction n: Pre- to post n: Pre- to FU**
**Self-reported measures**					
Pain intensity (VAS, range 0–10)	–1.4 (0.79)	–2.0 (0.88)	*F*_2__.__0__,__15__.__7_ = 3.12, *p* = 0.07, η*_*p*_*^2^ = 0.28	n.a.	6/10 5/9
Pain-related fear (PHODA; range 0–100, cutoff score 38)	–40.7 (5.6)	–37.3 (5.4)	*F*_(__1__.__4__,__10__.__0__)_ = 44.24, *p* < 0.001^∗∗^, η*_*p*_*^2^ = 0.86	Post- < Pre-EXP FU- < Pre-EXP	9/10 8/9
Fear of movement (TSK; range 17–68)	–12.2 (2.6)	–9.2 (3.6)	*F*_(__1__.__8__,__14__.__2__)_ = 8.49, *p* = 0.005^∗∗^, η*_*p*_*^2^ = 0.52	Post- < Pre-EXP	4/10 4/9
Pain catastrophizing (PCS; range 0–52, cutoff score 21)	–11.7 (5.5)	–11.2 (5.7)	*F*_(__1__.__1__,__8__.__5__)_ = 4.05, *p* = 0.08, η*_*p*_*^2^ = 0.34	n.a.	6/10 4/9
Pain disability (PDI; range 0–70)	–27.7 (4.2)	–25.6 (5.6)	*F*_(__1__.__3__,__10__.__5__)_ = 24.84, *p* < 0.001^∗∗^, η*_*p*_*^2^ = 0.76	Post- < Pre-EXP FU- < Pre-EXP	10/10 7/9
Perceived activity decline (PAD; range 0–20)	–6.9 (1.7)	–6.8 (2.3)	*F*_(__1__.__7__,__12__.__0__)_ = 8.13, *p* = 0.007, η*_*p*_*^2^ = 0.54	Post- < Pre-EXP	7/10 6/8
Physical activity (PARS; range 0–100)	–8.0 (3.3)	–4.4 (4.8)	*F*_(__1__.__6__,__13__.__0__)_ = 2.05, *p* = 0.17, η*_*p*_*^2^ = 0.20	n.a.	4/10 3/9
**Performance tasks**					
Two-min walking test (distance in meter)	42.9 (8.7)	44.5 (13.6)	*F*_(__1__.__2__,__7__.__1__)_ = 12.42, *p* = 0.008, η*_*p*_*^2^ = 0.67	Post- < Pre-EXP FU- < Pre-EXP	n.a.
Stair case walking (average time per step in seconds)	0.57 (0.12)	0.53 (0.13)	*F*_(__1__.__1__,__5__.__3__)_ = 17.82, *p* = 0.007, η*_*p*_*^2^ = 0.78	Post- < Pre-EXP FU- < Pre-EXP	n.a.

**^∗∗^Survives Bonferroni correction for multiple comparison (alpha = 0.05/9 = 0.0055), n.a., not applicable.**

##### Pre- to post changes in controls

Controls did not show any effects of Time (all *p*’s > 0.05).

##### Group effects post-EXP

Post-EXP, groups did not differ anymore in fear of movement, pain catastrophizing, self-reported physical activity, and staircase walking. Patients still reported higher pain intensity and pain-related disability compared to controls, and performed significantly worse on the 2 min walking test (Table 6).

**TABLE 6:** Self-reported measures and performance tasks post-EXP.

	**Post-EXP**			**FU-EXP**
	**Patients with cLBP Mean (SD)**	**Pain-free volunteers Mean (SD)**	**Statistics for group comparison**	**Patients with cLBP Mean (SD)**
**Self-reported measures**				
Pain intensity (VAS)	3.2 (2.9)	0.2 (0.3)	*F*_(__1__,__17__)_ = 9.91, *p* = 0.006, η*_*p*_*^2^ = 0.37, 95% CI = 1.0, 5.1	2.8 (2.9)
Pain-related fear (PHODA)	10.1 (16.2)	n.a.^+^	n.a.	16.0 (19.4)
Fear of movement (TSK)	28.9 (7.6)	29.3 (3.7)	*F*_(__1__,__17__)_ = 0.02, *p* = 0.88, η*_*p*_*^2^ = 0.001, 95% CI = -6.3, 5.5	32.8 (6.4)
Pain catastrophizing (PCS)	9.0 (9.7)	3.1 (3.3)	*F*_(__1__,__17__)_ = 3.00, *p* = 0.10, η*_*p*_*^2^ = 0.15, 95% CI = -1.3, 13.1	10.1 (9.6)
Pain disability (PDI)	9.4 (6.9)	1.0 (1.7)	*F*_(__1__,__17__)_ = 12.73, *p* = 0.002^∗∗^, η*_*p*_*^2^ = 0.42, 95% CI = 3.4, 13.4	11.9 (12.9)
Perceived activity decline (PAD)	3.4 (4.2)	1.1 (3.3)	*F*_(__1__,__17__)_ = 1.69, *p* = 0.21, η*_*p*_*^2^ = 0.09, 95% CI = -1.4, 6.0	4.3 (4.1)
Physical activity (PARS)	44.3 (10.9)	46.9 (6.5)	*F*_(__1__,__17__)_ = 0.38, *p* = 0.55, η*_*p*_*^2^ = 0.02, 95% CI = -11.4, 6.3	41.9 (13.9)
**Performance tasks**				
Two-min walking test (distance in meters)	201.6 (27.8)	231.4 (29.9)	*F*_(__1__,__16__)_ = 4.79, *p* = 0.04, η*_*p*_*^2^ = 0.23, 95% CI = -58.8, -0.94	193.9 (23.2)
Stair case walking (average time per step in seconds)	0.81 (0.21)	0.64 (0.14)	*F*_(__1__,__15__)_ = 3.48, *p* = 0.08, η*_*p*_*^2^ = 0.19, 95% CI = -0.02, 0.35	0.83 (0.26)

**^∗∗^Survives Bonferroni correction for multiple comparison (alpha = 0.05/9 = 0.0055); ^+^Due to a technical error, responses were not recorded for the majority of volunteers, n.a., not applicable; 95% CI, 95% confidence interval of the difference (patients – controls).**

#### Patients Report Less Fear for MOVEMENT Pictures After EXP Treatment

##### Pre- to post- to FU-EXP changes in patients

**FIGURE 5:**
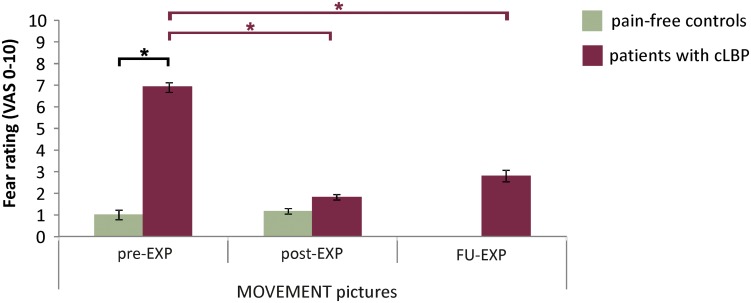
EXP treatment-induced changes in fear ratings. Presented are the means and standard errors for the MOVEMENT pictures for each group across time. Horizontal lines and asterisks indicate significant effects (^∗^*p* < 0.05): group effects are shown in black, while simple effects of Session, separate per group are shown in color (red for patients with cLBP; there were no significant Session effects for controls).

There was a significant effect of Time for fear ratings for the MOVEMENT pictures [*F*_(__1__.__56__,__12__.__44__)_ = 24.76, *p* < 001, η*_*p*_*^2^ = 0.76], with a significant decrease in ratings between pre- and post-EXP (*p-corr* < 0.001, 95% CI = –7.0, –3.2) and between pre- and FU-EXP (*p-corr* = 0.006, 95% CI = –7.2, –1.4), but no difference between post-EXP and FU-EXP (*p-corr* = 0.81, 95% CI = –1.3, 2.9) (Figure 5 and Supplementary Figure S3 ratings across all Picture Categories).

##### Pre- to post changes in controls

There was no significant effect of Time [*F*_(__1__,__9__)_ = 0.31, *p* = 0.59, η*_*p*_*^2^ = 0.03].

##### Group effects post-EXP

There was a significant Time x Group interaction [*F*_(__1__,__18__)_ = 55.20, *p* < 0.001, η*_*p*_*^2^ = 0.78]. Simple effects per time point showed that at post-EXP, there was no longer a Group difference [*F*_(__1__,__18__)_ = 1.12, *p* = 0.30, η*_*p*_*^2^ = 0.06, 95% CI = –1.9, 0.6].

#### Patients Show a Decrease in BOLD Activation to MOVEMENT Pictures After EXP Treatment

##### Pre- to post- to FU-EXP changes in patients

The effect of Time was investigated in the clusters showing a group difference pre-treatment (extracted betas from right posterior insula and mPFC clusters) as well as in a whole-brain analysis and in the predefined ROI masks.

**FIGURE 6:**
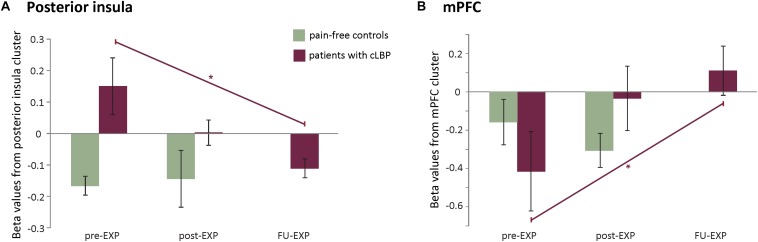
EXP treatment-induced effects in neural activation to MOVEMENT pictures in the posterior insula **(A)** and mPFC **(B)**. Plotted are averaged beta values and standard errors per time point and per group. Purple lines and asterisks indicate the significant linear effects over Time in patients. ^∗^*p* < 0.05.

The posterior insula cluster showed a main effect of Time [*F*_(__1__.__8__,__14__.__8__)_ = 4.06, *p* = 0.04, η*_*p*_*^2^ = 0.34], explained by a linearly decreasing response to MOVEMENT pictures over Time [*F*_(__1__,__8__)_ = 7.02, *p* = 0.03, η*_*p*_*^2^ = 0.40]. The mPFC only showed a marginally significant main effect of Time [*F*_2__.__0__,__158_ = 3.25, *p* = 0.07, η*_*p*_*^2^ = 0.29], explained by linearly increasing response to MOVEMENT pictures over Time [*F*_(__1__,__8__)_ = 8.7878, *p* = 0.02, η*_*p*_*^2^ = 0.41] (see Figure 6).

**FIGURE 7:**
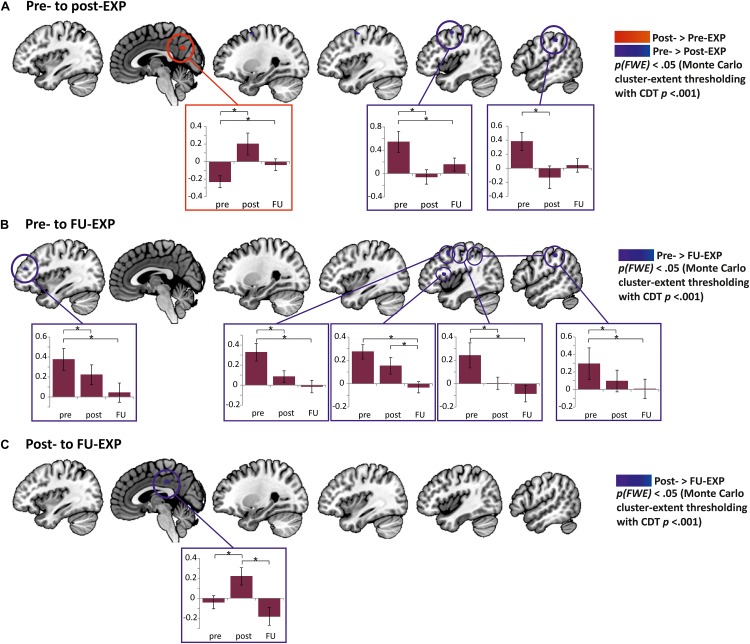
Clusters of EXP treatment-induced changes in neural activation to MOVEMENT pictures in patients with cLBP. **(A)** Differences from pre- to post-EXP were observed in precuneus (increase, red) as well as precentral gyrus and postcentral gyrus/supramarginal gyrus (from left to right; both decreases, blue). In the boxes, the extracted betas from the corresponding cluster are presented seperately for pre-, post- and FU-EXP. Significant differences across Sessions are highlighted by an asterisk (*p* < 0.05). **(B)** Differences from pre- to FU-EXP along with corresponding beta plots. Significant changes were observed in left middle frontal gyrus, right middle frontal/dorsolateral PFC, right inferior frontal/ventrolateral PFC, right postcentral gyrus, and right angular/inferior parietal lobe (from left to right, all decreases, blue). **(C)** Differences from post- to FU-EXP changes along with corresponding beta plots. A significant difference was found in the posterior cingulate cortex (PCC, decrease, blue). Cluster-level correction using *p* < 0.001 as initial threshold. Presented in the boxes are means and standard errors. CDT, cluster-defining threshold.

The whole-brain analyses showed a decrease in right post-central/supramarginal gyrus and pre-central gyrus, and an increase in activity in the precuneus from pre- to post-treatment (Figure 7 and Table 7). Comparing pre-treatment to 6 months follow-up, the right angular/inferior parietal lobe, right post-central, right middle frontal/dorsolateral PFC, right inferior frontal/ventrolateral PFC as well as left middle frontal gyrus showed a significant decrease in activation. Lastly, from post-treatment to 6 months follow-up, the right posterior cingulate cortex showed an additional decrease in activation. When evaluating the effect of Time in the predefined ROIs, there was a significant decrease from pre- to FU-EXP in the NAc (Table 7), but not in the other ROIs.

**TABLE 7:** EXP-induced changes in neural activation to MOVEMENT pictures.

		**MNI**			
		
		***x***	***y***	***z***	**Cluster size**
**Patients: pre- to post-EXP (whole-brain analysis – minimum cluster size 202 mm^3^)**					
R postcentral gyrus/inferior parietal lobe	Pre > Post	54	–28	53	285
R precentral gyrus	Pre > Post	42	–1	60	738
R Precuneus	Pre < Post	6	–62	60	436
**Patients: pre- to FU-EXP**					
**Whole-brain analysis (minimum cluster size 210 mm^3^)**					
R inferior parietal lobe	Pre > FU	54	–31	50	495
R postcentral gyrus	Pre > FU	45	–16	47	228
R middle frontal gyrus/dlPFC	Pre > FU	42	8	50	586
R inferior frontal gyrus/vlPFC	Pre > FU	45	11	13	292
L middle frontal gyrus	Pre > FU	–33	47	22	347
**Masked region of interest analysis (FDR *q* < 0.05)**					
Nucleus accumbens	Pre > FU	–15	17	–5	4
**Patients: post- to FU-EXP (whole-brain analysis – minimum cluster size 159 mm^3^)**					
R/L posterior cingulate gyrus	Post > FU	3	–28	38	208

##### Pre- to post changes in controls.

There were no effects of Time in the posterior insula and mPFC cluster. In controls, the whole-brain analysis revealed a change in two regions that do not overlap with the regions identified in patients (Supplementary Table S4). None of the predefined ROIs showed an effect of Time.

#### BOLD Activation to MOVEMENT Pictures Does Not Differ Anymore Between Patients and Controls After EXP Treatment

##### Group effects post-EXP

Post-treatment, no group differences were present anymore in the whole-brain analysis (also not when being less conservative with an initial threshold of *p* < 0.005 for cluster-size thresholding). None of the predefined ROIs showed a group difference post-EXP. In addition, when performing a Group comparison of the extracted betas from these ROIs, no group difference was identified at post-EXP [posterior insula: *F*_(__1__,__18__)_ = 2.58, *p* = 0.13, η*_*p*_*^2^ = 0.13, 95% CI = −0.38, 0.05; mPFC: *F*_(__1__,__18__)_ = 2.11, *p* = 0.16, η*_*p*_*^2^ = 0.11, 95% CI = −0.12, 0.63].

#### Neural Activation Changes to MOVEMENT Pictures in Patients Correlate With Changes in Fear Ratings (Explorative Analyses)

**FIGURE 8:**
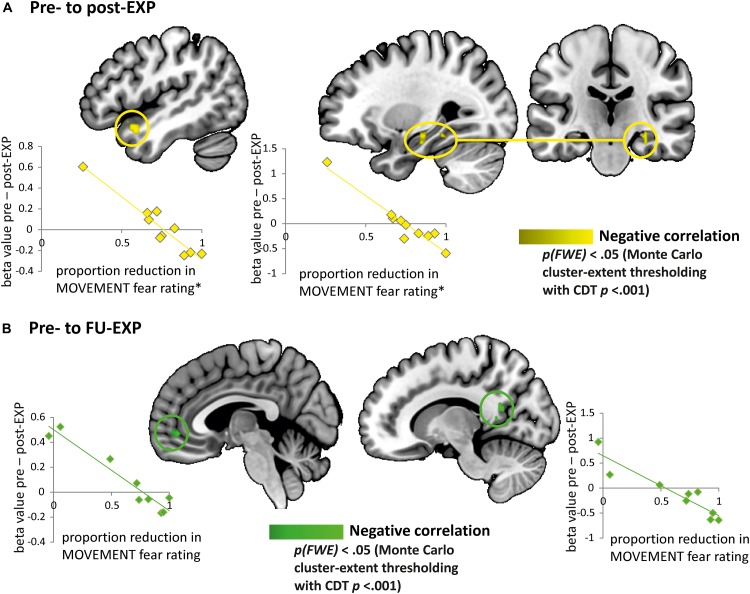
Explorative analyses: EXP treatment-induced changes in fear correlate with changes in neural responses to pain-related fear. **(A)** Brain regions showing a correlation between change in fear rating and change in neural activation (beta value) from pre- to post-EXP, corresponding to the left temporal pole and the left hippocampus (yellow). The scatterplots present the correlations between the change in neural activation in temporal pole (left) and hippocampus (right) with the proportion of reduction in fear ratings from pre- to post-EXP. ^∗^Note that the correlations were evaluated with and without the outlier (i.e., the individual with the lowest reduction in MOVEMENT fear rating). The outlier was not influential, as the correlations were still highly significant. **(B)** Brain regions showing a correlation between change in fear rating and change in neural activation (beta value) from pre- to FU-EXP, corresponding to the ventromedial prefrontal cortex (vmPFC)/anterior cingulate cortex (ACC) and the posterior cingulate cortex (PCC) (green). The scatterplots present the correlations between the change in neural activation in vmPFC (left) and PCC (right) with the proportion of reduction in fear ratings from pre- to FU-EXP. Cluster-level correction using *p* < 0.005 as initial cluster-defining threshold (CDT).

We explored whether changes in fear ratings for the MOVEMENT pictures from pre- to post-treatment were associated with specific changes in BOLD activation from pre- to post-treatment in patients. We found indications that a decrease in fear ratings from pre- to post-treatment was correlated to an increase of neural activation in the right hippocampus (MNI *x* = 30, *y* = –22, *z* = –17, *k* = 396 mm^3^) and the left temporal pole (MNI *x* = –42, *y* = 14, *z* = –20, *k* = 568 mm^3^) (see Figure 8). When extracting beta values, we found that the increase in BOLD activation in both regions was also related to decreases in pain-related fear from pre- to post-EXP (PHODA; left hippocampus: *r* = –0.82, *p* = 0.003, temporal pole: *r* = –0.89, *p* = 0.001) and to decreases in pain-related disability from pre- to post-EXP (PDI; left hippocampus: *r* = –0.78, *p* = 0.007, temporal pole: *r* = –0.71, *p* = 0.02).

The decrease in fear ratings from pre- to FU-EXP was furthermore related to an increase in right PCC (MNI *x* = 6, *y* = –55, *z* = 10, *k* = 407 mm^3^) and mPFC (MNI *x* = 0, *y* = 47, *z* = –5, *k* = 564 mm^3^). The right PCC betas additionally showed significant correlations to decreases in pain-related fear from pre- to FU-EXP (PHODA; *r* = –0.88, *p* = 0.002). Figure 8 shows these relations in more detail.

None of these clusters showed a main effect of Time [hippocampus: *F*_(__2__.__0__,__15__.__7__)_ = 0.35, *p* = 0.71, η*_*p*_*^2^ = 0.04; temporal pole: *F*_(__1__.__6__,__12__.__5__)_ = 0.03, *p* = 0.94, η*_*p*_*^2^ = 0.004; PCC: *F*_(__1__.__7__,__13__.__9__)_ = 2.87, *p* = 0.10, η*_*p*_*^2^ = 0.26; mPFC: *F*_(__1__.__5__,__12__.__1__)_ = 0.29, *p* = 0.69, η*_*p*_*^2^ = 0.04].

## Discussion

We provide the first evidence that clinical improvements following EXP in patients with cLBP are mirrored by changes in the neural circuitry for pain-related fear, the main target of EXP. Pre-treatment, we identified group differences in in-scanner fear ratings and neural responses to pictures of back-specific movements: compared to pain-free controls, patients with cLBP showed increased activation in the right posterior insula and increased deactivation in mPFC. Post-treatment, group differences were no longer present, and the process of change continued in patients at 6 months follow-up. Apart from general changes across treatment in lateral PFC, PCC, precuneus, NAc, and pre- and post-central gyrus, patients showed neural changes specifically related to changes in in-scanner fear ratings in the temporal pole, mPFC, PCC, and hippocampus. Pain-free volunteers did not show this, indicating that these changes cannot be attributed to general habituation effects. Hence, we provide evidence for treatment-induced neural changes in chronic pain that are specific to and correlate with improvements in self-reported fear.

### Replicating the Positive Clinical Effects of EXP

As expected, after EXP treatment, pain-related fear and disability significantly decreased while the patient’s performance (i.e., walking and stair case walking) improved significantly. Changes were maintained, or in some cases even more pronounced, 6 months after the end of treatment. We did not observe a significant effect of EXP on pain intensity, which is not uncommon nor unexpected. EXP focuses on reducing pain-related disabilities and reducing pain intensity is no explicit aim. Some studies, however, have observed significant improvements in pain intensity on a group level (den Hollander et al., 2016; Glombiewski et al., 2018), and also in the current study we observed improvements in some patients (i.e., clinically meaningful reduction in 60% of the patients). In future studies, it would be interesting to examine why some people respond with a reduction in pain intensity, while others do not. The lack of effect on pain catastrophizing is surprising though and not expected, given previous studies (see e.g., Leeuw et al., 2008; den Hollander et al., 2016; Lopez-de-Uralde-Villanueva et al., 2016) and the focus of EXP on disconfirming negative beliefs (Vlaeyen et al., 2012; den Hollander et al., 2015). Also for pain catastrophizing, however, we did observe a reduction on average as well as clinically meaningful reductions in 60% of patients (pre to post-EXP), suggesting that there was an effect which did not reach significance due to a relatively small sample size.

### Pre-treatment Group Differences in Fear Circuitry

We identified two brain regions showing a group difference in neural responses to pain-related fear. In the right posterior insula and mPFC, patients with cLBP showed altered neural activation compared to controls in response to our fear-evoking task. Focusing on pain-related fear, previous studies have demonstrated increased activation in the insula, as well as in other in regions including the ACC, superior parietal cortex, amygdala, orbitofrontal cortex, and striatum in patients compared to controls (Taylor et al., 2015; Meier et al., 2016). A potential explanation for the difference in extent of findings is our more stringent statistical thresholding (Woo et al., 2014) (i.e., with less stringent parameters, additional brain regions showed group differences; and when taking the picture categories together, a multitude of regions differed across groups, including ACC, superior parietal cortex and striatum, see Supplementary Information). Previous work related activation in insula, amygdala and several other regions to the amount of pain-related fear (Meier et al., 2016). Here, we extend these findings by showing that increased posterior insula activation is furthermore related to pain-related disability and actual physical performance (i.e., walking). In addition, its response was parametrically modulated by in-scanner fear ratings (Supplementary Information), further strengthening its specific involvement in pain-related fear. The insula is a core region involved in fear learning (Sehlmeyer et al., 2009; Fullana et al., 2016, 2018b), although loci are typically more anterior. The posterior insula, in contrast, has been associated with interoceptive integration (Craig, 2002), sensory aspects of pain/nociception (Garcia-Larrea and Peyron, 2013; Wager et al., 2013; Segerdahl et al., 2015), and experimental rather than clinical pain (Schweinhardt and Bushnell, 2010). This fits with abundant connections between posterior insula and somatosensory cortex (SI/SII; Wiech et al., 2014). Our finding that posterior insula activation was modulated by fear ratings, however, indicates additional involvement in pain-related fear, possibly due to a top-down modulatory effect of fear on this more sensory region.

The mPFC, and more specifically its ventromedial part (vmPFC), is also a core region involved in fear acquisition and extinction (Sehlmeyer et al., 2009), and general emotion regulation (Sotres-Bayon et al., 2006; Hartley and Phelps, 2010). mPFC involvement in pain and chronic pain is furthermore extensive (Ong et al., 2018). Our finding that mPFC showed a decreased (i.e., increased deactivation) response to fear-evoking stimuli in patients could point toward altered inhibitory control, and reduced ability to modulate or self-regulate pain (Tracey, 2010; Woo et al., 2015; Ong et al., 2018). To our surprise, amygdala activation to feared stimuli was not different across groups. Previous studies consistently reported the amygdala as a brain area of interest in (chronic) pain (see e.g., Simons et al., 2012) and fear or more generally threat (LeDoux, 1993). It may be that functional connectivity rather than neural activation distinguishes patients from controls. This will have to be explored in further analyses.

### Patient-Specific Neural Changes Across Treatment

The increased posterior insula response to our stimuli in patients pre-treatment was reduced over the course of EXP, as was the increased mPFC deactivation. Importantly, we no longer observed group differences post-treatment. This is in accordance with normalizations observed in fear ratings as well as in most clinical measures. Treatment effects were still present or even increased at 6 months follow-up, suggesting generalization to daily life. This is in accordance with a recent RCT in complex regional pain syndrome, where EXP effect sizes were larger at 6 months follow up compared to post-treatment (den Hollander et al., 2016).

Furthermore, several brain regions showed changes in neural responses across treatment, including pre- and post-central gyrus/supramarginal gyrus, precuneus, lateral PFC, and NAc. In *pre- and post-central gyrus/supramarginal gyrus*, we observed decreases from pre- to post-EXP and from pre-EXP to follow-up. Recruitment of these areas associated with motor control, sensory properties of somatosensory stimuli (Peyron et al., 2000), as well as sensorimotor imagery (McNorgan, 2012; Hetu et al., 2013) was expected, as participants were imagining performing movements and activities depicted in the stimuli. Functional changes in sensorimotor regions have previously been identified in chronic pain (Flodin et al., 2014; Kregel et al., 2015). The changes over time we observed may reflect normalizations in sensorimotor neurocircuitry, and along similar lines it may also reflect changes in physical performance that go alongside with EXP, as an indirect result of reducing pain-related fear. The *precuneus*, on the other hand, showed increased activation over the course of treatment. The precuneus is part of the default-mode network (DMN), involved in interoception, mentalizing, integrating information more than processing it (Cavanna and Trimble, 2006). Its activation has been negatively correlated to pain sensitivity, without contributing to the actual neural representation of pain (Goffaux et al., 2014), the direction of which is in line with our findings. Interestingly, in fibromyalgia, abnormalities in connectivity between the insula (including posterior part) and the DMN have been observed (Napadow et al., 2010), and changes herein and in posterior insula glutamate levels have been observed following treatment-induced pain reductions (Napadow et al., 2012; Harris et al., 2013). *Two prefrontal clusters*, one in dorsal, one in ventral lateral PFC and a subcortical *NAc* cluster showed decreased activation from pre-EXP to 6 months follow-up. The NAc is a major reward center of the brain, and has been implicated in the regulation of pain (Woo et al., 2015) and in the chronification of pain (Baliki et al., 2012; Borsook et al., 2016). It is also associated with experiencing pain in the chronic phase (Hashmi et al., 2013), representing its motivational value. Our finding indicates that EXP also induces changes in the motivational component of pain and associated pain-related cues (e.g., reduced motivational salience of the back-related pictures following EXP). The dlPFC is also involved in the regulation of pain (Lorenz et al., 2003; Seminowicz and Moayedi, 2017), and abnormally increased activation has been observed in chronic pain (Seminowicz and Moayedi, 2017). Interestingly, following treatment, activation in the dlPFC during a cognitively demanding task as well as increases in cortical thickness were normalized (Seminowicz et al., 2011). In contrast, the vlPFC has been associated with affective/motivational processing, and control of goal-directed behavior (Taylor et al., 2004; Sakagami and Pan, 2007). It has extensive connections with orbitofrontal cortex and subcortical areas such as the amygdala, and also interacts with motor regions to orient attention (Corbetta and Shulman, 2002). Neural changes in this region to pain stimuli have been observed following CBT in fibromyalgia, but in opposite directions (Jensen et al., 2012). Importantly, additional analyses show that such changes did not occur in controls (Supplementary Information), suggesting that these time-dependent changes are not due to general habituation effects, but instead specific to the patient group and likely attributable to treatment.

### Neural Changes Specific to Reductions in Pain-Related Fear Ratings

We explored whether fear reduction was associated with specific changes in neural activation to our stimuli. In these explorative analyses, we found indications that pre- to post-EXP decreases in fear ratings were associated with neural activation increases in right hippocampus and left temporal pole. Decreased ratings from pre-EXP to follow-up were associated with increases in the mPFC and PCC. The mPFC, PCC, and hippocampus are associated with fear extinction (Sehlmeyer et al., 2009). Reduced hippocampal volumes and abnormal hippocampal connectivity have been reported in chronic pain (Mutso et al., 2012, 2013). Treatment-induced increases in mPFC neural activation in relation to decreases in fear is in agreement with increased inhibitory control occurring during fear extinction. Cautiously, our findings suggest that extinction during EXP may reflect similar working mechanisms as observed during experimental extinction studies. Noted, the initial cluster-defining statistical threshold (CDT) for cluster-size thresholding was slightly less conservative (*p* < 0.005), which we consider fair given the additional constraints of the analysis. Also note that these regions did not show main effects across treatment, suggesting individual rather than group-level differences. Future analyses will have to investigate whether there are functional connectivity alterations between mPFC and amygdala, which would be the hypothesized mechanism of extinction (Phelps et al., 2004; but also see Fullana et al., 2018a; Morriss et al., 2018).

### Limitations and Future Considerations

Our findings should be interpreted in light of its limitations. First, there was no control treatment, hence we cannot infer that neural changes are specific to EXP. Though, our pain-free control group did control for effects of practice and time. And as we focused on pain-related fear -the main target of EXP-, related findings to within-session fear ratings as well as to clinical assessments of pain-related outcomes, this adds to the specificity of our findings. Second, the focus here is on the MOVEMENT category, because it is most relevant for our patient group, but also for simplicity reasons. Not all findings were specific to this category (e.g., the other two categories also showed pre-EXP posterior insula differences). However, most importantly, time-dependent changes in these regions were specific to this category (Supplementary Information). Finally, the relatively small sample size may have comprimised our statistical power, and motivated us to focus on the whole-brain correlation analysis only (i.e., no other correlations with changes over time), limiting the generalizability of our findings. Several participants could not be included in our analyses or were lost to follow-up, partly because our study was conducted amidst clinical standard care (e.g., the patient and/or clinical team decided not to start EXP), and partly due to the challenges of conducting MRI research in clinical pain populations. Despite that, we show strong data of group differences as well as changes across time, all surviving stringent statistical testing. Larger samples will be needed to reproduce the current findings, and to extend to models predicting treatment responses.

## Conclusion

We show the first evidence that clinical improvements in chronic pain following EXP treatment are mirrored by changes in pain-related fear neural circuitry. Group differences identified prior to treatment were no longer present after treatment. Time-dependent effects in patients continued up to 6 months after the end of EXP, and involved regions implicated in cognitive/affective, motivational as well as sensory aspects related to pain. This suggests that the effects of EXP are long-term and go above and beyond modulating fear circuitry. Lastly, explorative analyses found indications that brain regions implicated in fear extinction -including the hippocampus, PCC and mPFC- changed their neural response proportionate to the change in self-reported fear, suggesting that extinction during EXP may reflect similar working mechanisms as extinction in experimental settings. Taken together, our findings show that neural circuitry for pain-related fear is modulated by EXP, and that changes are associated with self-reported improvements in pain-related fear.

## Data Availability

The datasets generated for this study are available on reasonable request to the corresponding author.

## Ethics Statement

This study involving human participants was reviewed and approved by the Medical Ethical Committee of Maastricht University Hospital/Maastricht University (MUMC+/UM). All participants provided their written informed consent to participate in this study.

## Author Contributions

IT, JJ, MG, JV, RS, and AK contributed to the conception and design of the study. IT and AK acquired the data and contributed to the data analysis plan. IT performed the data analysis and wrote the manuscript. All authors contributed to the manuscript, and read and approved the final version.

## Conflict of Interest Statement

The authors declare that the research was conducted in the absence of any commercial or financial relationships that could be construed as a potential conflict of interest.

## Footnotes

1 http://www.qualtrics.com

2 Also, participants were asked not to change anything in their medication use on the day of the MRI, and all confirmed they did not take less or more medication than usual. See Supplementary Table S3 for an overview of the patients’ medication use.

## References

[B1] Baliki M. N. Geha P. Y. Jabakhanji R. Harden N. Schnitzer T. J. Apkarian A. V. (2008). A preliminary fMRI study of analgesic treatment in chronic back pain and knee osteoarthritis. *Mol. Pain* 4:47. 10.1186/1744-8069-4-47 18950528 PMC2584040

[B2] Baliki M. N. Petre B. Torbey S. Herrmann K. M. Huang L. Schnitzer T. J. (2012). Corticostriatal functional connectivity predicts transition to chronic back pain. *Nat. Neurosci.* 15 1117–1119. 10.1038/nn.3153 22751038 PMC3411898

[B3] Becerra L. Sava S. Simons L. E. Drosos A. M. Sethna N. Berde C. (2014). Intrinsic brain networks normalize with treatment in pediatric complex regional pain syndrome. *Neuroimage Clin.* 6 347–369. 10.1016/j.nicl.2014.07.012 25379449 PMC4218937

[B4] Boersma K. Linton S. Overmeer T. Jansson M. Vlaeyen J. De Jong J. (2004). Lowering fear-avoidance and enhancing function through exposure in vivo. A multiple baseline study across six patients with back pain. *Pain* 108 8–16. 10.1016/j.pain.2003.03.001 15109502

[B5] Borsook D. Linnman C. Faria V. Strassman A. M. Becerra L. Elman I. (2016). Reward deficiency and anti-reward in pain chronification. *Neurosci. Biobehav. Rev.* 68 282–297. 10.1016/j.neubiorev.2016.05.033 27246519

[B6] Bosma R. L. Cheng J. C. Rogachov A. Kim J. A. Hemington K. S. Osborne N. R. (2018). Brain dynamics and temporal summation of pain predicts neuropathic pain relief from ketamine infusion. *Anesthesiology* 129 1015–1024. 10.1097/ALN.0000000000002417 30199420

[B7] Breivik H. Collett B. Ventafridda V. Cohen R. Gallacher D. (2006). Survey of chronic pain in Europe: prevalence, impact on daily life, and treatment. *Eur. J. Pain* 10 287–333. 16095934 10.1016/j.ejpain.2005.06.009

[B8] Bunzli S. Smith A. Schutze R. O’sullivan P. (2015). Beliefs underlying pain-related fear and how they evolve: a qualitative investigation in people with chronic back pain and high pain-related fear. *BMJ Open* 5:e008847. 10.1136/bmjopen-2015-008847 26482773 PMC4611881

[B9] Camacho-Soto A. Sowa G. A. Perera S. Weiner D. K. (2012). Fear avoidance beliefs predict disability in older adults with chronic low back pain. *PM R* 4 493–497. 10.1016/j.pmrj.2012.01.017 22516436 PMC3917604

[B10] Cavanna A. E. Trimble M. R. (2006). The precuneus: a review of its functional anatomy and behavioural correlates. *Brain* 129 564–583. 10.1093/brain/awl004 16399806

[B11] Corbetta M. Shulman G. L. (2002). Control of goal-directed and stimulus-driven attention in the brain. *Nat. Rev. Neurosci.* 3 201–215. 10.1038/nrn755 11994752

[B12] Craig A. D. (2002). How do you feel? Interoception: the sense of the physiological condition of the body. *Nat. Rev. Neurosci.* 3 655–666. 10.1038/nrn894 12154366

[B13] Crombez G. Vlaeyen J. W. S. Heuts P. H. Lysens R. (1999). Pain-related fear is more disabling than pain itself: evidence on the role of pain-related fear in chronic back pain disability. *Pain* 80 329–339. 10.1016/s0304-3959(98)00229-2 10204746

[B14] de Jong J. R. Vangronsveld K. Peters M. L. Goossens M. E. J. B. Onghena P. Bulté I. (2008). Reduction of pain-related fear and disability in post-traumatic neck pain: a replicated single-case experimental study of exposure in vivo. *J. Pain* 9 1123–1134. 10.1016/j.jpain.2008.06 18722818

[B15] de Jong J. R. Vlaeyen J. W. S. Onghena P. Goossens M. E. J. B. Geilen M. Mulder H. (2005). Fear of movement/(re)injury in chronic low back pain: education or exposure in vivo as mediator to fear reduction? *Clin. J. Pain* 21 9–17. 10.1097/00002508-200501000-00002 15599127

[B16] de Jong J. R. Vlaeyen J. W. S. Van Eijsden M. Loo C. Onghena P. (2012). Reduction of pain-related fear and increased function and participation in work-related upper extremity pain (WRUEP): effects of exposure in vivo. *Pain* 153 2109–2118. 10.1016/j.pain.2012.07.001 22902198

[B17] den Hollander M. Goossens M. E. J. B. De Jong J. R. Ruijgrok J. Oosterhof J. Onghena P. (2016). Expose or protect? A randomized controlled trial of exposure in vivo versus physiotherapy in patients with complex regional pain syndrome type 1. *Pain* 157 2318–2329. 10.1097/j.pain.0000000000000651 27429174

[B18] den Hollander M. Meulders A. Jakobs M. Vlaeyen J. W. (2015). The effect of threat information on acquisition, extinction, and reinstatement of experimentally conditioned fear of movement-related pain. *Pain Med.* 16 2302–2315. 10.1111/pme.12836 26360518

[B19] Desikan R. S. Segonne F. Fischl B. Quinn B. T. Dickerson B. C. Blacker D. (2006). An automated labeling system for subdividing the human cerebral cortex on MRI scans into gyral based regions of interest. *Neuroimage* 31 968–980. 10.1016/j.neuroimage.2006.01.021 16530430

[B20] Erpelding N. Simons L. Lebel A. Serrano P. Pielech M. Prabhu S. (2014). Rapid treatment-induced brain changes in pediatric CRPS. *Brain Struct. Funct.* 221 1095–1111. 10.1007/s00429-014-0957-8 25515312 PMC4470894

[B21] Flodin P. Martinsen S. Lofgren M. Bileviciute-Ljungar I. Kosek E. Fransson P. (2014). Fibromyalgia is associated with decreased connectivity between pain- and sensorimotor brain areas. *Brain Connect.* 4 587–594. 10.1089/brain.2014.0274 24998297 PMC4202907

[B22] Frazier J. A. Chiu S. Breeze J. L. Makris N. Lange N. Kennedy D. N. (2005). Structural brain magnetic resonance imaging of limbic and thalamic volumes in pediatric bipolar disorder. *Am. J. Psychiatry* 162 1256–1265. 10.1176/appi.ajp.162.7.1256 15994707

[B23] Fullana M. A. Albajes-Eizagirre A. Soriano-Mas C. Vervliet B. Cardoner N. Benet O. (2018a). Amygdala where art thou? *Neurosci. Biobehav. Rev.* 102 430–431. 29886178 10.1016/j.neubiorev.2018.06.003

[B24] Fullana M. A. Albajes-Eizagirre A. Soriano-Mas C. Vervliet B. Cardoner N. Benet O. (2018b). Fear extinction in the human brain: a meta-analysis of fMRI studies in healthy participants. *Neurosci. Biobehav. Rev.* 88 16–25. 10.1016/j.neubiorev.2018.03.002 29530516

[B25] Fullana M. A. Harrison B. J. Soriano-Mas C. Vervliet B. Cardoner N. Avila-Parcet A. (2016). Neural signatures of human fear conditioning: an updated and extended meta-analysis of fMRI studies. *Mol. Psychiatry* 21 500–508. 10.1038/mp.2015.88 26122585

[B26] Garcia-Larrea L. Peyron R. (2013). Pain matrices and neuropathic pain matrices: a review. *Pain* 154 (Suppl.) S29–S43. 10.1016/j.pain.2013.09.001 24021862

[B27] GBD 2015 Disease and Injury Incidence and Prevalence Collaborators (2016). Global, regional, and national incidence, prevalence, and years lived with disability for 310 diseases and injuries, 1990-2015: a systematic analysis for the global burden of disease study 2015. *Lancet* 388 1545–1602. 10.1016/S0140-6736(16)31678-6 27733282 PMC5055577

[B28] Glombiewski J. A. Holzapfel S. Riecke J. Vlaeyen J. W. S. De Jong J. Lemmer G. (2018). Exposure and CBT for chronic back pain: an RCT on differential efficacy and optimal length of treatment. *J. Consult. Clin. Psychol.* 86 533–545. 10.1037/ccp0000298 29781651

[B29] Goffaux P. Girard-Tremblay L. Marchand S. Daigle K. Whittingstall K. (2014). Individual differences in pain sensitivity vary as a function of precuneus reactivity. *Brain Topogr.* 27 366–374. 10.1007/s10548-013-0291-0 23636269

[B30] Goldstein J. M. Seidman L. J. Makris N. Ahern T. O’brien L. M. Caviness V. S. (2007). Hypothalamic abnormalities in schizophrenia: sex effects and genetic vulnerability. *Biol. Psychiatry* 61 935–945. 10.1016/j.biopsych.2006.06.027 17046727

[B31] Harris R. E. Napadow V. Huggins J. P. Pauer L. Kim J. Hampson J. (2013). Pregabalin rectifies aberrant brain chemistry, connectivity, and functional response in chronic pain patients. *Anesthesiology* 119 1453–1464. 10.1097/ALN.0000000000000017 24343290

[B32] Hartley C. A. Phelps E. A. (2010). Changing fear: the neurocircuitry of emotion regulation. *Neuropsychopharmacology* 35 136–146. 10.1038/npp.2009.121 19710632 PMC3055445

[B33] Hartvigsen J. Hancock M. J. Kongsted A. Louw Q. Ferreira M. L. Genevay S. (2018). What low back pain is and why we need to pay attention. *Lancet* 391 2356–2367. 10.1016/S0140-6736(18)30480-X 29573870

[B34] Hashmi J. A. Baliki M. N. Huang L. Baria A. T. Torbey S. Hermann K. M. (2013). Shape shifting pain: chronification of back pain shifts brain representation from nociceptive to emotional circuits. *Brain* 136 2751–2768. 10.1093/brain/awt211 23983029 PMC3754458

[B35] Hetu S. Gregoire M. Saimpont A. Coll M. P. Eugene F. Michon P. E. (2013). The neural network of motor imagery: an ALE meta-analysis. *Neurosci. Biobehav. Rev.* 37 930–949. 10.1016/j.neubiorev.2013.03.017 23583615

[B36] Icenhour A. Langhorst J. Benson S. Schlamann M. Hampel S. Engler H. (2015). Neural circuitry of abdominal pain-related fear learning and reinstatement in irritable bowel syndrome. *Neurogastroenterol. Motil.* 27 114–127. 10.1111/nmo.12489 25557224

[B37] Jensen K. B. Kosek E. Wicksell R. Kemani M. Olsson G. Merle J. V. (2012). Cognitive behavioral therapy increases pain-evoked activation of the prefrontal cortex in patients with fibromyalgia. *Pain* 153 1495–1503. 10.1016/j.pain.2012.04.010 22617632

[B38] Kattoor J. Gizewski E. R. Kotsis V. Benson S. Gramsch C. Theysohn N. (2013). Fear conditioning in an abdominal pain model: neural responses during associative learning and extinction in healthy subjects. *PLoS One* 8:e51149. 10.1371/journal.pone.0051149 23468832 PMC3582635

[B39] Kori S. H. (1990). Kinisophobia: a new view of chronic pain behavior. *Pain Manage* 3 35–43.

[B40] Kregel J. Meeus M. Malfliet A. Dolphens M. Danneels L. Nijs J. (2015). Structural and functional brain abnormalities in chronic low back pain: a systematic review. *Semin. Arthritis Rheum.* 45 229–237. 10.1016/j.semarthrit.2015.05.002 26092329

[B41] Labus J. S. Hubbard C. S. Bueller J. Ebrat B. Tillisch K. Chen M. (2013). Impaired emotional learning and involvement of the corticotropin-releasing factor signaling system in patients with irritable bowel syndrome. *Gastroenterology* 145 e1–e3. 10.1053/j.gastro.2013.08.016 23954313 PMC4069031

[B42] Lalouni M. Olen O. Bonnert M. Hedman E. Serlachius E. Ljotsson B. (2016). Exposure-Based cognitive behavior therapy for children with abdominal pain: a pilot trial. *PLoS One* 11:e0164647. 10.1371/journal.pone.0164647 27736943 PMC5063361

[B43] Lang P. J. Bradley M. M. Cuthbert B. N. (1997). *International Affective Picture System (IAPS): Technical Manual and Affective Ratings.* Gainesville, FL: NIMH Center for the Study of Emotion and Attention, 39–58.

[B44] LeDoux J. E. (1993). Emotional memory: in search of systems and synapses. *Ann. N. Y. Acad. Sci.* 702 149–157. 10.1111/j.1749-6632.1993.tb17246.x 8109874

[B45] Leeuw M. Goossens M. E. Van Breukelen G. J. Boersma K. Vlaeyen J. W. (2007). Measuring perceived harmfulness of physical activities in patients with chronic low back pain: the Photograph Series of Daily Activities–short electronic version. *J. Pain* 8 840–849. 10.1016/j.jpain.2007.05.013 17632038

[B46] Leeuw M. Goossens M. E. J. B. Van Breukelen G. J. P. De Jong J. R. Heuts P. H. T. G. Smeets R. J. E. M. (2008). Exposure in vivo versus operant graded activity in chronic low back pain patients: results of a randomized controlled trial. *Pain* 138 192–207. 10.1016/j.pain.2007.12.009 18242858

[B47] Linnman C. Moulton E. A. Barmettler G. Becerra L. Borsook D. (2012). Neuroimaging of the periaqueductal gray: state of the field. *Neuroimage* 60 505–522. 10.1016/j.neuroimage.2011.11.095 22197740 PMC3288184

[B48] Lopez-de-Uralde-Villanueva I. Munoz-Garcia D. Gil-Martinez A. Pardo-Montero J. Munoz-Plata R. Angulo-Diaz-Parreno S. (2016). A systematic review and meta-analysis on the effectiveness of graded activity and graded exposure for chronic nonspecific low back pain. *Pain Med.* 17 172–188. 26235368 10.1111/pme.12882

[B49] Lorenz J. Minoshima S. Casey K. L. (2003). Keeping pain out of mind: the role of the dorsolateral prefrontal cortex in pain modulation. *Brain* 126 1079–1091. 10.1093/brain/awg102 12690048

[B50] Makris N. Goldstein J. M. Kennedy D. Hodge S. M. Caviness V. S. Faraone S. V. (2006). Decreased volume of left and total anterior insular lobule in schizophrenia. *Schizophr. Res.* 83 155–171. 10.1016/j.schres.2005.11.020 16448806

[B51] McNeil D. W. Rainwater A. J. (1998). Development of the fear of pain questionnaire-III. *J. Behav. Med.* 21 389–410. 9789168 10.1023/a:1018782831217

[B52] McNorgan C. (2012). A meta-analytic review of multisensory imagery identifies the neural correlates of modality-specific and modality-general imagery. *Front. Hum. Neurosci.* 6:285. 10.3389/fnhum.2012.00285 23087637 PMC3474291

[B53] Meier M. L. Stampfli P. Humphreys B. K. Vrana A. Seifritz E. Schweinhardt P. (2017). The impact of pain-related fear on neural pathways of main modulation in chronic low back pain. *Pain Rep.* 2:e601. 10.1097/PR9.0000000000000601 29392216 PMC5741307

[B54] Meier M. L. Stampfli P. Vrana A. Humphreys B. K. Seifritz E. Hotz-Boendermaker S. (2016). Neural correlates of fear of movement in patients with chronic low Back Pain vs. Pain-Free individuals. *Front. Hum. Neurosci.* 10:386. 10.3389/fnhum.2016.00386 27507941 PMC4960248

[B55] Meulders A. Vlaeyen J. W. (2012). Reduction of fear of movement-related pain and pain-related anxiety: an associative learning approach using a voluntary movement paradigm. *Pain* 153 1504–1513. 10.1016/j.pain.2012.04.013 22617631

[B56] Morriss J. Hoare S. Van Reekum C. M. (2018). It’s time: a commentary on fear extinction in the human brain using fMRI. *Neurosci. Biobehav. Rev.* 94 321–322. 10.1016/j.neubiorev.2018.06.025 29969594

[B57] Mutso A. A. Petre B. Huang L. Baliki M. N. Torbey S. Herrmann K. M. (2013). Reorganization of hippocampal functional connectivity with transition to chronic back pain. *J. Neurophysiol.* 111 1065–1076. 10.1152/jn.00611.2013 24335219 PMC3949236

[B58] Mutso A. A. Radzicki D. Baliki M. N. Huang L. Banisadr G. Centeno M. V. (2012). Abnormalities in hippocampal functioning with persistent pain. *J. Neurosci.* 32 5747–5756. 10.1523/JNEUROSCI.0587-12.2012 22539837 PMC3365570

[B59] Napadow V. Kim J. Clauw D. J. Harris R. E. (2012). Decreased intrinsic brain connectivity is associated with reduced clinical pain in fibromyalgia. *Arthritis Rheum.* 64 2398–2403. 10.1002/art.34412 22294427 PMC3349799

[B60] Napadow V. Lacount L. Park K. As-Sanie S. Clauw D. J. Harris R. E. (2010). Intrinsic brain connectivity in fibromyalgia is associated with chronic pain intensity. *Arthritis Rheum.* 62 2545–2555. 10.1002/art.27497 20506181 PMC2921024

[B61] Ong W. Y. Stohler C. S. Herr D. R. (2018). Role of the Prefrontal Cortex in Pain Processing. *Mol. Neurobiol.* 56 1137–1166. 10.1007/s12035-018-1130-9 29876878 PMC6400876

[B62] Peyron R. Laurent B. Garcia-Larrea L. (2000). Functional imaging of brain responses to pain: a review and meta-analysis. *Neuropsychol. Clin.* 30 263–288. 10.1016/s0987-7053(00)00227-6 11126640

[B63] Phelps E. A. Delgado M. R. Nearing K. I. Ledoux J. E. (2004). Extinction learning in humans: role of the amygdala and vmPFC. *Neuron* 43 897–905. 15363399 10.1016/j.neuron.2004.08.042

[B64] Sakagami M. Pan X. (2007). Functional role of the ventrolateral prefrontal cortex in decision making. *Curr. Opin. Neurobiol.* 17 228–233. 10.1016/j.conb.2007.02.008 17350248

[B65] Schweinhardt P. Bushnell M. C. (2010). Pain imaging in health and disease — how far have we come ? *J. Clin. Investig.* 120 3788–3797. 10.1172/JCI43498 21041961 PMC2964988

[B66] Segerdahl A. R. Mezue M. Okell T. W. Farrar J. T. Tracey I. (2015). The dorsal posterior insula subserves a fundamental role in human pain. *Nat. Neurosci.* 18 499–500. 10.1038/nn.3969 25751532 PMC6783299

[B67] Sehlmeyer C. Schoning S. Zwitserlood P. Pfleiderer B. Kircher T. Arolt V. (2009). Human fear conditioning and extinction in neuroimaging: a systematic review. *PLoS One* 4:e5865. 10.1371/journal.pone.0005865 19517024 PMC2692002

[B68] Seminowicz D. Wideman T. H. Naso L. Hatami-Khoroushahi Z. Fallatah S. Ware M. (2011). Effective treatment of chronic low back pain in humans reverses abnormal brain anatomy and function. *J. Neurosci.* 31 7540–7550. 10.1523/JNEUROSCI.5280-10.2011 21593339 PMC6622603

[B69] Seminowicz D. A. Moayedi M. (2017). The dorsolateral prefrontal cortex in acute and chronic pain. *J. Pain* 18 1027–1035. 10.1016/j.jpain.2017.03.008 28400293 PMC5581265

[B70] Simons L. E. Moulton E. A. Linnman C. Carpino E. Becerra L. Borsook D. (2012). The human amygdala and pain: evidence from neuroimaging. *Hum. Brain Mapp.* 35 527–538. 10.1002/hbm.22199 23097300 PMC3920543

[B71] Simons L. E. Pielech M. Erpelding N. Linnman C. Moulton E. Sava S. (2014). The responsive amygdala: treatment-induced alterations in functional connectivity in pediatric complex regional pain syndrome. *Pain* 155 1727–1742. 10.1016/j.pain.2014.05.023 24861582 PMC4157948

[B72] Soer R. Koke A. J. Vroomen P. C. Stegeman P. Smeets R. J. Coppes M. H. (2013). Extensive validation of the pain disability index in 3 groups of patients with musculoskeletal pain. *Spine* 38 E562–E568. 10.1097/BRS.0b013e31828af21f 23388675

[B73] Sotres-Bayon F. Cain C. K. Ledoux J. E. (2006). Brain mechanisms of fear extinction: historical perspectives on the contribution of prefrontal cortex. *Biol. Psychiatry* 60 329–336. 10.1016/j.biopsych.2005.10.012 16412988

[B74] Spielberger C. D. Gorsuch R. L. Lushene P. R. Vagg P. R. Jacobs A. G. (1983). *Manual for the State-Trait Anxiety Inventory (Form Y).* Palo Alto, CA: Consulting Psychologists Press.

[B75] Sullivan M. J. Bishop S. R. Pivik J. (1995). The pain catastrophizing scale: development and validation. *Psychol. Assess.* 7 524–532. 10.1037//1040-3590.7.4.524

[B76] Tait R. C. Pollard C. A. Margolis R. B. Duckro P. N. Krause S. J. (1987). The pain disability index: psychometric and validity data. *Arch. Phys. Med. Rehabil.* 68 438–441. 3606368

[B77] Taylor A. M. Harris A. D. Varnava A. Phillips R. Taylor J. O. Hughes O. (2015). A functional magnetic resonance imaging study to investigate the utility of a picture imagination task in investigating neural responses in patients with chronic musculoskeletal pain to daily physical activity photographs. *PLoS One* 10:e0141133. 10.1371/journal.pone.0141133 26496709 PMC4619796

[B78] Taylor S. F. Welsh R. C. Wager T. D. Phan K. L. Fitzgerald K. D. Gehring W. J. (2004). A functional neuroimaging study of motivation and executive function. *Neuroimage* 21 1045–1054. 10.1016/j.neuroimage.2003.10.032 15006672

[B79] Thibodeau M. A. Fetzner M. G. Carleton R. N. Kachur S. S. Asmundson G. J. (2013). Fear of injury predicts self-reported and behavioral impairment in patients with chronic low back pain. *J. Pain* 14 172–181. 10.1016/j.jpain.2012.10.014 23260450

[B80] Tracey I. (2010). Getting the pain you expect: mechanisms of placebo, nocebo and reappraisal effects in humans. *Nat. Med.* 16 1277–1283. 10.1038/nm.2229 20948533

[B81] van der Ploeg H. M. Defares P. B. Spielberger C. D. Defares P. B. Spielberger C. D. (1980). *Handleiding bij de Zelf-Beoordelings Vragenlijst ZBV: een nederlandstalige bewerking van de Spielberger State-trait Anxiety Inventory STAI-DY. [Manual for the Self-Assessment Questionnaire ZBV: a Dutch-language adaptation of the Spielberger State-Trait Anxiety Inventory STAI-DY.].* Lisse: Swets & Zeitlinger.

[B82] van Wijk A. J. Hoogstraten J. (2006). Dutch translation of the fear of pain questionnaire: factor structure, reliability and validity. *Eur. J. Pain* 10 479–486. 16095936 10.1016/j.ejpain.2005.06.008

[B83] Verbunt J. A. (2008). Reliability and validity of the PAD questionnaire: a measure to assess pain-related decline in physical activity. *J. Rehabil. Med.* 40 9–14. 10.2340/16501977-0126 18176731

[B84] Vercoulen J. H. Bazelmans E. Swanink C. M. Fennis J. F. Galama J. M. Jongen P. J. (1997). Physical activity in chronic fatigue syndrome: assessment and its role in fatigue. *J. Psychiatr. Res.* 31 661–673. 9447571 10.1016/s0022-3956(97)00039-3

[B85] Vlaeyen J. W. Crombez G. Linton S. J. (2016). The fear-avoidance model of pain. *Pain* 157 1588–1589. 10.1097/j.pain.0000000000000574 27428892

[B86] Vlaeyen J. W. De Jong J. Geilen M. Heuts P. H. Van Breukelen G. (2001). Graded exposure in vivo in the treatment of pain-related fear: a replicated single-case experimental design in four patients with chronic low back pain. *Behav. Res. Ther.* 39 151–166. 10.1016/s0005-7967(99)00174-6 11153970

[B87] Vlaeyen J. W. Kole-Snijders A. M. Boeren R. G. Van Eek H. (1995a). Fear of movement/(re)injury in chronic low back pain and its relation to behavioral performance. *Pain* 62 363–372. 10.1016/0304-3959(94)00279-n 8657437

[B88] Vlaeyen J. W. S. Kole-Snijders A. M. Rotteveel A. M. Ruesink R. Heuts P. H. (1995b). The role of fear of movement/(re)injury in pain disability. *J. Occup. Rehabil.* 5 235–252. 10.1007/bf02109988 24234727

[B89] Vlaeyen J. W. Morley S. J. Linton S. J. Boersma K. De Jong J. (2012). *Pain-related Fear: Exposure-Based Treatment for Chronic Pain.* Seattle, WA: IASP Press.

[B90] Vlaeyen J. W. S. Crombez G. (1999). Fear of movement/(re)injury, avoidance and pain disability in chronic low back pain patients. *Man. Ther.* 4 187–195. 10.1054/math.1999.0199 10593107

[B91] Wager T. D. Atlas L. Y. Lindquist M. A. Roy M. Woo C.-W. Kross E. (2013). An fMRI-based neurologic signature of physical pain. *New Engl. J. Med.* 368 1388–1397. 10.1056/NEJMoa1204471 23574118 PMC3691100

[B92] Wiech K. Jbabdi S. Lin C. S. Andersson J. Tracey I. (2014). Differential structural and resting state connectivity between insular subdivisions and other pain-related brain regions. *Pain* 155 2047–2055. 10.1016/j.pain.2014.07.009 25047781 PMC4220010

[B93] Woo C. W. Krishnan A. Wager T. D. (2014). Cluster-extent based thresholding in fMRI analyses: pitfalls and recommendations. *Neuroimage* 91 412–419. 10.1016/j.neuroimage.2013.12.058 24412399 PMC4214144

[B94] Woo C. W. Roy M. Buhle J. T. Wager T. D. (2015). Distinct brain systems mediate the effects of nociceptive input and self-regulation on pain. *PLoS Biol.* 13:e1002036. 10.1371/journal.pbio.1002036 25562688 PMC4285399

[B95] Woods M. P. Asmundson G. J. (2008). Evaluating the efficacy of graded in vivo exposure for the treatment of fear in patients with chronic back pain: a randomized controlled clinical trial. *Pain* 136 271–280. 10.1016/j.pain.2007.06.037 17716819

[B96] Zale E. L. Lange K. L. Fields S. A. Ditre J. W. (2013). The relation between pain-related fear and disability: a meta-analysis. *J. Pain* 14 1019–1030. 10.1016/j.jpain.2013.05.005 23850095 PMC3791167

[B97] Zhu X. Suarez-Jimenez B. Lazarov A. Helpman L. Papini S. Lowell A. (2018). Exposure-based therapy changes amygdala and hippocampus resting-state functional connectivity in patients with posttraumatic stress disorder. *Depress. Anxiety* 35 974–984. 10.1002/da.22816 30260530 PMC6168398

