# Supplementary material for: Exposure in vivo Induced Changes in Neural Circuitry for Pain-Related Fear: A Longitudinal fMRI Study in Chronic Low Back Pain
Source: Front Neurosci. 2019 Sep 17;13:970. doi: 10.3389/fnins.2019.00970 (PMC6758595; doi:10.3389/fnins.2019.00970)
Supplement: Supplementary file 1 [file Data_Sheet_1.pdf]

Supplementary Information

Exposure in Vivo induced changes in neural circuitry for pain-related fear: a longitudinal fMRI study in chronic low back pain

Short title: Neural correlates of exposure treatment

Inge Timmers[1,2,3], Jeroen R de Jong[1,4,6], Mariëlle Goossens[1,5], Jeanine A Verbunt[1,4,6], Rob J Smeets[1,7], Amanda L Kaas[2]

[1] Department of Rehabilitation Medicine, Maastricht University, PO Box 616, 6200 MD, Maastricht, the Netherlands; [2] Department of Cognitive Neuroscience, Maastricht University, PO Box 616, 6200 MD, Maastricht, the Netherlands; [3] Department of Anesthesiology, Perioperative, and Pain Medicine, Stanford University, 1070 Arastradero Road, 94304 CA Palo Alto, United States; [4] Department of Rehabilitation Medicine, Maastricht University Medical Center, PO Box 5800, 6202 AZ, Maastricht, the Netherlands; [5] Department of Clinical Psychological Science, Maastricht University, PO Box 616, 6200 MD, Maastricht, the Netherlands; [6] Adelante Centre of Expertise in Rehabilitation and Audiology, Hoensbroek, The Netherlands; [7] CIR Revalidatie, Zwolle/Eindhoven, the Netherlands

Corresponding author: Inge Timmers, Department of Anesthesiology, Perioperative, and Pain Medicine, Stanford University, 1070 Arastradero Road, 94304 CA Palo Alto, United States, itimmers@stanford.edu

Supplementary Methods and Materials

Data analysis

Denoising of the MRI data. First the ratings were inspected per and across categories to identify outlier ratings[1]. This resulted in discarding a total of four REST picture trials, as well as twelve MEDICAL picture trials (on an individual subject level). Individual movement parameters were added as predictors of no interest: the six detrended motion parameters, the six first derivates of these parameters, and potential motion spikes (i.e., defined as motion surpassing a threshold of .25 mm of root mean square displacement from one volume to the next volume; using the Motion Correction Processor plugin in BrainVoyager). In case the motion exceeded the voxel size (3 mm), the participant was excluded from data analyses. Based on this criterion, three patients were excluded. In addition, time courses from the white matter and cerebral spinal fluid were extracted, averaged and normalized, and added as confounders of no interest as well. Designs with and without confounders were compared in terms of temporal signal to noise (tSNR) using the Model Tester plugin in BrainVoyager. For all participants, the design with all confounders resulted in the best tSNR. Data were inspected on an individual basis by contrasting all picture categories with baseline. Based on this, one additional patient was excluded due to lack of any vision-related (occipital) neural activity for viewing the pictures.

MRI data analysis: Parametric modulation by fear. For the patients, an additional analysis was the parametric modulation by fear at pre-EXP. An additional design matrix was created including three predictors coding for the parametric modulation of the HRF by the individual fearfulness ratings (REST_parametric, MOVEMENT_parametric, MEDICAL_parametric). Parametric modulation was examined by the conjunction contrast of the main predictor and parametric predictor for the MOVEMENT condition. The parametric modulation analysis was run in the patient group only, since the controls were expected to report very low fear levels for this condition (with very little variation). Also for this analysis, an initial threshold of $p < .001$ was used after which a cluster-size thresholding was performed using MonteCarlo simulations (n=1000) to correct maps at the level of alpha 0.05 at the whole brain level. Masked region of interest analyses were performed too. In these masks, q(FDR) < .05 and a minimum cluster size of 4 voxels (108 mm^3) was used for statistical thresholding.

---

[1] An outlier was defined as follows: for REST pictures, a rating was considered an outlier if it was a rating of 7.5 or higher and a rating higher compared to average of all MEDICAL pictures for that participant. For MEDICAL pictures, a rating was considered an outlier if it was a rating of 2.5 or lower and a rating lower compared to the average of all REST pictures of that participant.

Supplementary Results

Pre-treatment (pre-EXP) data

Patients report more fear for all Picture Categories pre-EXP

The ratings collected during the picture imagination run showed a significant Picture Category x Group interaction effect [$F_{1.9, 49.4} = 37.44$, $p < .001$], as well as significant main effects of Picture Category [$F_{1.9, 49.4} = 142.45$, $p < .001$] and Group [$F_{1, 26} = 116.45$, $p < .001$], see Figure S1. Simple effects per group showed that there was a main effect of Picture Category in both groups [controls: $F_{1.1, 14.1} = 91.79$, $p < .001$, patients: $F_{1.4, 18.1} = 88.51$, $p < .001$]. In controls, there was a difference for all three possible comparisons between categories [all *p-corr* < .05]. In patients, REST pictures were rated as significantly less fearful than both MOVEMENT and MEDICAL fearful pictures [both *p-corr* < .05], while no difference was observed between MOVEMENT and MEDICAL fearful pictures [*p-corr* = .91]. Simple effects per Picture Category showed that the Groups differed in all three categories [REST: $F_{1, 26} = 73.38$, $p < .001$, MOVEMENT: $F_{1, 26} = 188.15$, $p < .001$; MEDICAL: $F_{1, 26} = 19.74$, $p < .001$].

When correlating the fear ratings with the full measure for pain-related fear (PHODA), correlations are significant for the MOVEMENT [$r = .64$, $p = .01$] and the MEDICAL pictures [$r = .63$, $p = .02$], but not for the REST pictures [$r = .47$, $p = .09$].

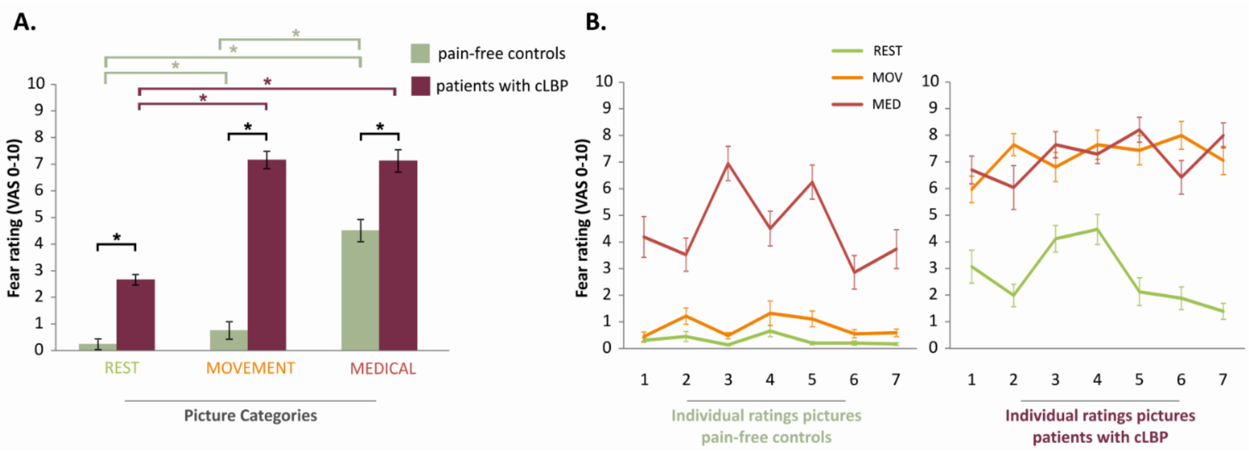

Figure S1. Fear ratings of the presented pictures. A. Fear ratings per Picture Category per Group. B. Fear ratings per Picture, separated for patients and controls. As can be observed in this figure, the MOVEMENT pictures were most distinguishing between the groups. * $p < .05$

Regions showing group differences display parametric modulation by fear ratings pre-EXP for MOVEMENT pictures only

MOVEMENT pictures. To inspect which brain regions are related to pain-related fear, we examined in which brain regions the activation was parametrically modulated by the self-reported fear to the MOVEMENT pictures. At the whole brain level, no clusters survived the cluster-

threshold correction of the conjunction effect (MOVEMENT main effect ∩ MOVEMENT parametric modulation effect). When inspecting the predefined ROIs as well as the clusters showing a group difference in MOVEMENT pictures, it was observed that the right posterior insula showed this conjunction effect (FDR-corrected within the ROI). Thus, this region showed both a main effect to the MOVEMENT picture as well as a parametric effect, indicating that the neural response (HRF) is modulated by the individual fear ratings. None of the other ROIs showed this effect.

Other Picture Categories. No parametric modulation effects were found at the whole brain level, nor in any of the ROIs for REST and for MEDICAL pictures.

More general group differences in neural activation to all Picture Categories combined

Figure S2 shows activation maps for all three Picture Categories separately, per group, as well as a main effect of Group (collapsed across all categories). As can be observed, when taking all Picture Categories together, there were a multitude of brain regions showing a significant difference across groups (see also Table S1).

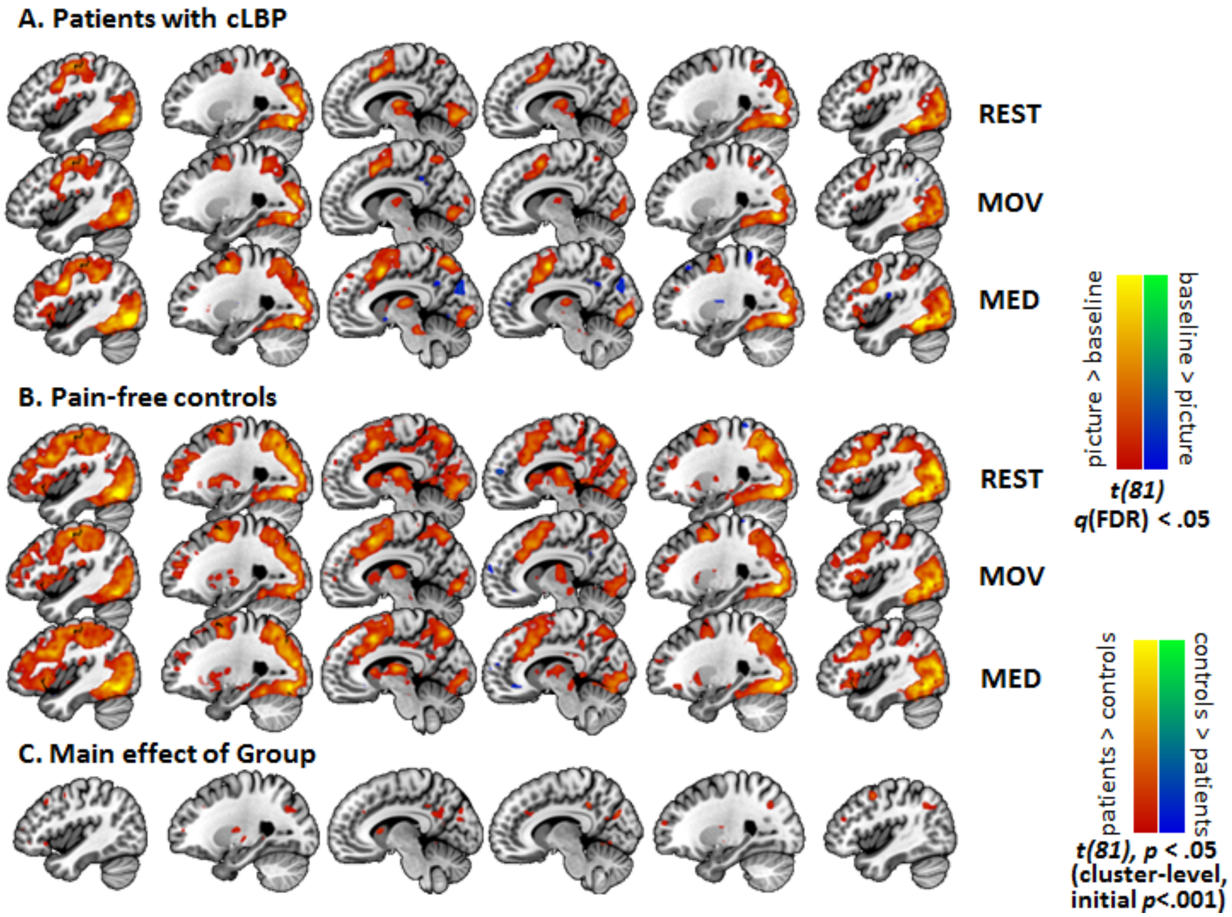

Figure S2. Activation maps for the different Picture Categories at pre-EXP, per group. Statistical maps are presented showing the neural activation of all categories relative to baseline for A. patients with cLBP, and B. pain-free volunteers, C. group differences across all three conditions.

Table S1. Overview of main effects of Group, showing group differences between patients and controls, collapsed over all three picture categories. Presented are coordinates of peak values per cluster (bold) as well as local maxima. *q*(FDR) < .05

			MNI coordinates			
Anatomical location			x	y	z	# voxels
L	**Inferior Frontal Gyrus**		-51	17	-5	27
R	**Inferior Frontal Gyrus**		33	32	-5	13
L	**Middle Frontal Gyrus**		-39	41	-8	16
L	**Middle Frontal Gyrus**		-30	29	34	15
L	**Middle Frontal Gyrus**		-36	23	49	31
L		Precentral Gyrus	-39	26	40	14
L	**Middle Frontal Gyrus**		-33	50	10	19
L	**Middle Frontal Gyrus**		-36	-1	46	16
R	**Middle Frontal Gyrus**		39	38	19	14
R	**Middle Frontal Gyrus**		39	20	25	17
R	**Superior Frontal Gyrus**		27	59	16	27
R		Middle Frontal Gyrus	36	56	7	12
L	**Insula**		-30	26	-5	9
R	**Claustrum**		33	-10	4	127
R		Insula	39	-1	13	8
R		Claustrum	33	-16	-5	26
R		Claustrum	36	-1	1	25
R		Lentiform Nucleus	21	-7	-5	9
L	**Medial Frontal Gyrus**		0	38	46	26
L	**Medial Frontal Gyrus**		0	23	46	17
L	**Medial Frontal Gyrus**		0	35	34	64
R		Cingulate Gyrus	6	26	28	14
R	**Cingulate Gyrus (posterior)**		6	-43	37	44
R		Cingulate Gyrus	6	-43	37	27
L		Cingulate Gyrus	-9	-43	37	17
R	**Precentral Gyrus (MI)**		45	5	49	34
R	**Precentral Gyrus (MI)**		54	-7	25	22
L	**Postcentral Gyrus (SI)**		-45	-22	40	10

L	**Postcentral Gyrus (SI)**	-63	-13	22	33
L	Postcentral Gyrus	-63	-13	31	9
R	**Postcentral Gyrus (SI)**	51	-13	49	31
R	Precentral Gyrus	54	-7	40	8
R	**Postcentral Gyrus (SI)**	36	-25	49	8
R	**Postcentral Gyrus (SI)**	57	-7	13	15
L	**Supramarginal Gyrus**	-51	-52	34	29
L	Supramarginal Gyrus	-60	-52	31	11
L	**Superior Parietal Lobe**	-33	-61	55	10
R	**Superior Parietal Lobe**	30	-55	46	12
R	**Inferior Parietal Lobe**	45	-55	40	55
R	Precuneus	42	-67	37	14
R	**Inferior Parietal Lobe**	54	-34	49	45
R	Inferior Parietal Lobe	51	-37	40	23
L	**Precuneus**	-6	-73	52	10
L	**Precuneus**	-21	-67	31	37
L	Superior Occipital Gyrus	-27	-76	31	12
L	**Precuneus**	0	-61	19	135
L	Cuneus	-3	-61	34	23
L	Posterior Cingulate	-12	-61	16	21
L	Cingulate Gyrus	-6	-52	28	42
L	Posterior Cingulate	-6	-49	19	10
R	**Precuneus**	24	-64	40	33
R	Precuneus	24	-73	40	13
L	**Cuneus**	-9	-82	34	24
L	Cuneus	-9	-79	22	12
R	**Cuneus**	9	-76	28	24
R	**Cuneus**	27	-91	-2	11
L	**Middle Temporal Gyrus**	-60	-46	-2	8
L	**Middle Temporal Gyrus**	-51	-34	-5	13
L	**Middle Temporal Gyrus**	-36	-73	34	9
R	**Middle Temporal Gyrus**	42	-67	13	14
L	**Superior Temporal Gyrus**	-66	-22	1	26
L	Superior Temporal Gyrus	-57	-22	1	8
R	**Middle Occipital Gyrus**	24	-88	25	15
L	**Culmen (cerebellum)**	-12	-52	-8	19
R	**Culmen (cerebellum)**	12	-58	-8	29
R	Lingual Gyrus	12	-70	1	9
L	**Lentiform Nucleus**	-27	-10	10	57

L		Lentiform Nucleus	-27	-19	1	28
L	**Caudate**		-9	14	7	33
L		Lentiform Nucleus	-18	17	7	12
L	**Parahippocampal Gyrus**		-24	-40	1	15
L		Parahippocampal Gyrus	-15	-37	-2	8

Groups also differ in several regions with respect to the other Picture Categories

When inspecting group difference at a whole-brain level for REST and MEDICAL pictures, several clusters are identified (see Table S2).

Table S2. Group differences in REST, MOVEMENT and MEDICAL at pre-EXP (whole brain analyses)

	MNI coordinates			
	x	y	z	Cluster size
REST (minimum cluster size 211 mm^3)*				
**R Angular Gyrus**	51	-34	46	452
**L Caudate**	-18	17	7	371
**R Caudate**	18	17	10	258
MOVEMENT (minimum cluster size 189 mm^3)*				
**R Posterior Insula**	33	-10	10	206
MEDICAL (minimum cluster size 190 mm^3)*				
**L Lingual**	-12	-52	-8	229

*Cluster-level correction using $p < .001$ as initial threshold.

Group differences in the other Pictures Categories in regions showing group difference in MOVEMENT pictures

To check the specificity of the group differences, we extracted the betas from the two clusters showing the group difference in MOVEMENT pictures (posterior insula, mPFC) and examined group difference in these regions for REST and MEDICAL pictures. For the posterior insula, the group difference was present as well in both other categories [REST pictures: $F_{1,26} = 5.45$, $p = .03$; MEDICAL pictures: $F_{1,26} = 10.63$, $p = .003$]. For the mPFC, there were no differences in the other categories [REST pictures: $F_{1,26} = 2.38$, $p = .14$; MEDICAL pictures: $F_{1,26} = 0.04$, $p = .84$].

Effects of Exposure in Vivo treatment

Medication use over the course of treatment

Table S3: Reported medication use on the day of the study visit

	Pre-EXP	Post-EXP	FU-EXP
**P10**	none	none	none
**P12**	none	n.a.	n.a.
**P13**	none	none	none
**P17**	anti-convulsant (pregabalin), SNRI (duloxetine)	anti-convulsant (pregabalin), SNRI (duloxetine)	n.a.
**P19**	none	none	none
**P20**	none	none	none
**P22**	none	none	TCA, opioid (tramadol), acetaminophen
**P24**	NSAID (ibuprofen), acetaminophen	n.a.	n.a.
**P26**	anti-convulsant (pregabalin), acetaminophen	none	none
**P27**	none	none	none
**P30**	benzodiazepine	benzodiazepine	benzodiazepine
**P34**	none	none	none
**P37**	none	n.a.	n.a.
**P39**	acetaminophen, opioid (morphine patch*)	n.a.	n.a.

n.a. = not applicable (did not participate in post-/FU-EXP); SNRI = serotonin and norepinephrine reuptake inhibitors; NSAID = nonsteroidal anti-inflammatory drugs; TCA = tricyclic antidepressants; *patch was not on during MRI scan

Correlations with changes in pain-related fear assessment are strongest for MOVEMENT pictures

Correlations between percentage reduction in pain-related fear as assessed using the PHODA and percentage reductions fear ratings were calculated for the patient group. From pre- to post-EXP as well as from pre- to FU-EXP, correlations were highest for the MOVEMENT pictures [pre- to post-EXP - REST pictures: $r = .53$, $p = .11$, MOVEMENT pictures: $r = .91$, $p < .001$, MEDICAL

pictures: $r = .33$, $p = .36$; pre- to FU-EXP - REST pictures: $r = .78$, $p = .01$, MOVEMENT pictures: $r = .83$, $p = .006$, MEDICAL pictures: $r = .55$, $p = .12$].

Fear ratings for the other Picture Categories change over time as well in the patient group

Pre- to post- to FU-EXP changes in patients: There was a significant Time x Picture Category interaction for the fear ratings [$F_{2.8,22.8} = 10.33$, $p < .001$] (see Figure S3). Simple effects per Picture Category shows that for REST pictures, patients showed a significant effect of Time [$F_{1.6,13.1} = 27.34$, $p < .001$], where there was a decrease in ratings from pre-EXP to post-/FU-EXP [*p-corr* < .05], but no difference between post-EXP and FU-EXP [*p-corr* > .05]. For MOVEMENT pictures a similar pattern emerged: a significant effect of Time [$F_{1.6,12.4} = 24.76$, $p < .001$], where ratings decreased from pre-EXP to post-/FU-EXP [*p-corr* < .05], but no difference was found between post-EXP and FU-EXP [*p-corr* > .05]. For MEDICAL pictures, a significant effect of Time was found as well [$F_{1.7,13.4} = 7.99$, $p = .007$]. Here, only a significant reduction was found from pre- to post-EXP [*p-corr* < .05].

Pre- to post changes in controls: For controls, no significant Time x Picture Category was observed [$F_{1.2,11.0} = 2.34$, $p = .15$], nor a main effect of Time [$F_{1,9} = 1.72$, $p = .22$]. Only Picture Category showed a main effect [$F_{1.2,10.4} = 68.66$, $p < .001$], with post-hoc comparisons showing that all Picture Categories differed significantly from each other [*p-corr* < .05].

Group effects pre- to post-EXP: There was a three-way interaction with Time x Picture Category x Group [$F_{1.7,31.5} = 11.67$, $p < .001$]. Simple effects per time point showed that at pre-EXP there was a significant Picture Category x Group [$F_{1.9,49.4} = 37.44$, $p < .001$] interaction, explained by the fact that controls gave different ratings in all three Picture Categories [*p-corr* < .05], while patients rated MOVEMENT and MEDICAL pictures equally high [*p-corr* > .05]. In addition, the patients gave higher ratings compared to controls in all three Picture Categories [REST: $F_{1,26} = 73.38$, $p < .001$; MOVEMENT: $F_{1,26} = 188.15$, $p < .001$; MEDICAL: $F_{1,26} = 19.74$, $p < .001$]. At post-EXP, no Picture Category x Group interaction was observed [$F_{1.5,26.4} = .55$, $p = .53$]. Here, only a main effect of Picture Category was found [$F_{1.5,26,5} = 41.24$, $p < .001$] with a significant difference across all three Picture Categories [*p-corr* < .05]. At post-EXP, no significant main effects of Group were observed [$F_{1,18} = .38$, $p = .55$].

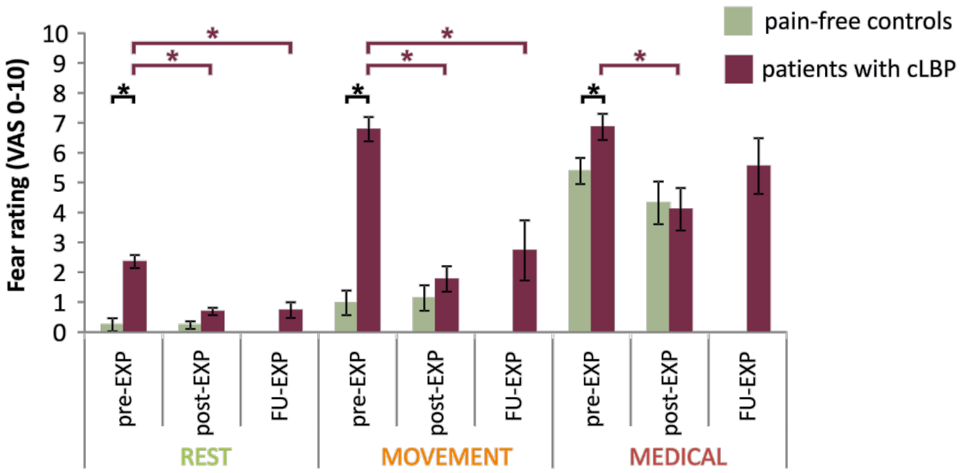

Figure S3. EXP treatment-induced changes in fear ratings of the presented pictures. Presented are the mean fear ratings for the Picture Categories: REST, MOVEMENT, and MEDICAL. Bars and asterisks indicate significant effects. In black, Group effects are shown. Simple effects of Session separate per group are indicated in purple (patients with cLBP; there were no significant Session effects for controls). Effects for Picture Category are not displayed here for clarity purposes. Presented are means and standard errors (SE). * $p < .05$

Changes in neural activation to MOVEMENT pictures for in controls

Pre- to post changes in controls: To exclude the possibility of a general effect of time or practice, we performed an analysis of Time in the control group. The right posterior insula, nor the mPFC showed an effect of Time [right posterior insula: $F_{1,9} = .07$, $p = .80$; mPFC: $F_{1,9} = 2.54$, $p = .15$].

Taking a more explorative whole brain level approach, two regions showed a significant change in activation at the second scan compared to the first: the left parietal operculum and left middle temporal gyrus (see Table S4). Both regions showed an increase in activation over time. These regions did not show any changes over time in the patient group (tested using a rmGLM on the extracted betas; main effect of Time parietal operculum: $F_{1.5,11.9} = 2.61$, $p = .12$; middle temporal gyrus: $F_{1.8,14.5} = 1.58$, $p = .24$].

Table S4. EXP-induced changes in neural activation to MOVEMENT pictures (controls)

		MNI			
		x	y	z	Cluster size
Controls: Pre- to post EXP (minimum cluster size 156 mm³)*					
**L Parietal Operculum**	Pre < Post	-45	-38	28	179
**L Middle Temporal Gyrus**	Pre < Post	-60	-55	-5	284

* Cluster-level correction using $p < .001$ as initial threshold.

No effects of Time in posterior insula and mPFC for the other Picture Categories

Effects of Time were examined in the clusters showing a group difference pre-treatment (extracted betas from right posterior insula and mPFC clusters). There were no effects of Time in posterior insula [REST pictures - main effect of Time: $F_{1.3,10.5} = 0.51$, $p = .54$, linear effect: $F_{1.,8} = 2.68$, $p = .14$; MEDICAL pictures - main effect of Time: $F_{1.8,14.1} = 0.47$, $p = .61$, linear effect: $F_{1.,8} = 1.01$, $p = .34$], nor in the mPFC [REST pictures - main effect of Time: $F_{1.9,14.9} = 0.62$, $p = .54$, linear effect: $F_{1.,8} = 0.95$, $p = .36$; MEDICAL pictures - main effect of Time: $F_{1.2,9.7} = 0.13$, $p = .77$, linear effect: $F_{1.,8} = 0.25$, $p = .63$]. A visual overview can be found in Figure S4.

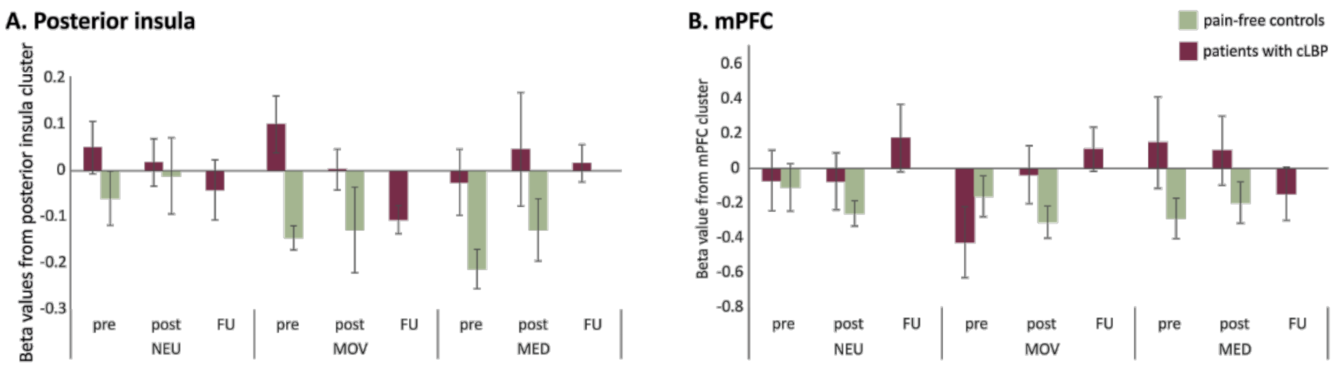

Figure S4. EXP treatment-induced effects in neural activation to all picture categories. Presented are averaged beta values and standard errors per picture category, group and time point, in the posterior insula (A) and mPFC (B) cluster.
